# A proof-of-concept pipeline to guide evaluation of tumor tissue perfusion by dynamic contrast-agent imaging: Direct simulation and inverse tracer-kinetic procedures

**DOI:** 10.3389/fbinf.2023.977228

**Published:** 2023-04-13

**Authors:** Irene E. Vignon-Clementel, Nick Jagiella, Jules Dichamp, Jérôme Kowalski, Wiltrud Lederle, Hendrik Laue, Fabian Kiessling, Oliver Sedlaczek, Dirk Drasdo

**Affiliations:** ^1^ Inria, Palaiseau, France; ^2^ Institute for Experimental Molecular Imaging (ExMI), University Clinic and Helmholtz Institute for Biomedical Engineering, RWTH Aachen University, Aachen, Germany; ^3^ Fraunhofer MEVIS, Institute for Digital Medicine, Bremen, Germany; ^4^ Fraunhofer MEVIS, Institute for Digital Medicine, Aachen, Germany; ^5^ Department of NCT Radiology Uniklinikum/DKFZ Heidelberg, Heidelberg, Germany; ^6^ IfADo - Leibniz Research Centre for Working Environment and Human Factors, Dortmund, Germany

**Keywords:** tumor perfusion, *in silico* imaging, tracer-kinetics models, microcirculation, dynamic contrast-enhanced imaging, parameter estimation

## Abstract

Dynamic contrast-enhanced (DCE) perfusion imaging has shown great potential to non-invasively assess cancer development and its treatment by their characteristic tissue signatures. Different tracer kinetics models are being applied to estimate tissue and tumor perfusion parameters from DCE perfusion imaging. The goal of this work is to provide an *in silico* model-based pipeline to evaluate how these DCE imaging parameters may relate to the true tissue parameters. As histology data provides detailed microstructural but not functional parameters, this work can also help to better interpret such data. To this aim *in silico* vasculatures are constructed and the spread of contrast agent in the tissue is simulated. As a proof of principle we show the evaluation procedure of two tracer kinetic models from *in silico* contrast-agent perfusion data after a bolus injection. Representative microvascular arterial and venous trees are constructed *in silico*. Blood flow is computed in the different vessels. Contrast-agent input in the feeding artery, intra-vascular transport, intra-extravascular exchange and diffusion within the interstitial space are modeled. From this spatiotemporal model, intensity maps are computed leading to *in silico* dynamic perfusion images. Various tumor vascularizations (architecture and function) are studied and show spatiotemporal contrast imaging dynamics characteristic of *in vivo* tumor morphotypes. The Brix II also called 2CXM, and extended Tofts tracer-kinetics models common in DCE imaging are then applied to recover perfusion parameters that are compared with the ground truth parameters of the *in silico* spatiotemporal models. The results show that tumor features can be well identified for a certain permeability range. The simulation results in this work indicate that taking into account space explicitly to estimate perfusion parameters may lead to significant improvements in the perfusion interpretation of the current tracer-kinetics models.

## 1 Introduction

Certain histological features have been found to be characteristic for classification of tissue pathologies, in particular the grading of solid tumors and their evolution in absence of or in response to treatment (Noguchi et al., 2008; Hu et al., 2009; Li and Padhani, 2012; Essock-Burns et al., 2013), hence their knowledge can impact on clinical decisions. These features can be quantified by parameters such as the density and type of cells, the density, morphology and the spatial architecture formed by the vessels, which may also provide indirect information on leakiness (e.g. depending on fenestration of vessels etc.). However, histological information is not readily accessible unless biopsies are taken, which, besides being invasive, only provide local, and mainly morphological, information. For this reason, it is valuable to infer information on tissue microarchitecture from macroscopic imaging modalities, ideally complemented by functional signatures like the vessels’ flow and leakiness. Specific imaging modalities such as dynamic contrast-enhanced (DCE) perfusion imaging, or diffusion weighted imaging (DW)-MRI have been developed to gather information about such parameters down to the histology level non-invasively (Palmowski et al., 2008b; Lassau et al., 2011; O’Connor et al., 2011). As these modalities do not typically give direct access to the set of microscopic parameters, the signal measured with a given non-invasive imaging modality needs to be related to the information of interest at the histological level. This includes the spatial micro-architecture, as usually the signal corresponds to an average over many micro-structural elements, and the functional information as, for example, vessel leakiness, given that knowing the localisation of a blood vessel wall alone is insufficient to infer its permeability. Consequently, these non-invasive modalities, if correctly applied and interpreted, have the potential to provide histological and functional information, and, due to their non-invasiveness, can be applied during the development and treatment of the disease making them potentially very valuable for clinical decisions. For DW-MRI, the signal information can, under certain conditions, be easily related to the quantitative local tumour density (Yin et al., 2018) and even be probed by biopsies (Yin et al., 2021). For DCE imaging, numerous tracer kinetics (TK) models (e.g. Brix, Tofts, … ) have been constructed such that a small set of model parameters is believed to reflect the information of interest for the vasculature, and hence permit to retrieve this information from the imaging signal (DCE-MRI, DCE-CT, DCE-US).However, validation of these TK models remains a great challenge. Structural parameters can be compared to invasive histological data (Zwick et al., 2009). Functional parameters can be included for validation with *in vitro* phantom experiments (Driscoll et al., 2011; Gauthier et al., 2012). However to construct a representative vascularised tumor phantom at scale is challenging and difficult to adapt to different micro-architectures. Comparison to other blood flow imaging measurement modalities have validated flow (Lee et al., 2003), but only at the macro-scale. An alternative could be a “virtual” validation: by setting up a spatial temporal model that, even if it represents a simplification of the tissue micro-architecture, captures the important parameters and features of a tissue at the histological level, and then testing at least *in silico*, in how far TK-models can be expected to infer information on the parameters of interest, potentially proposing inference strategies that are expected to improve the inference of microscopic information from macroscopic signals.

**FIGURE 1:**
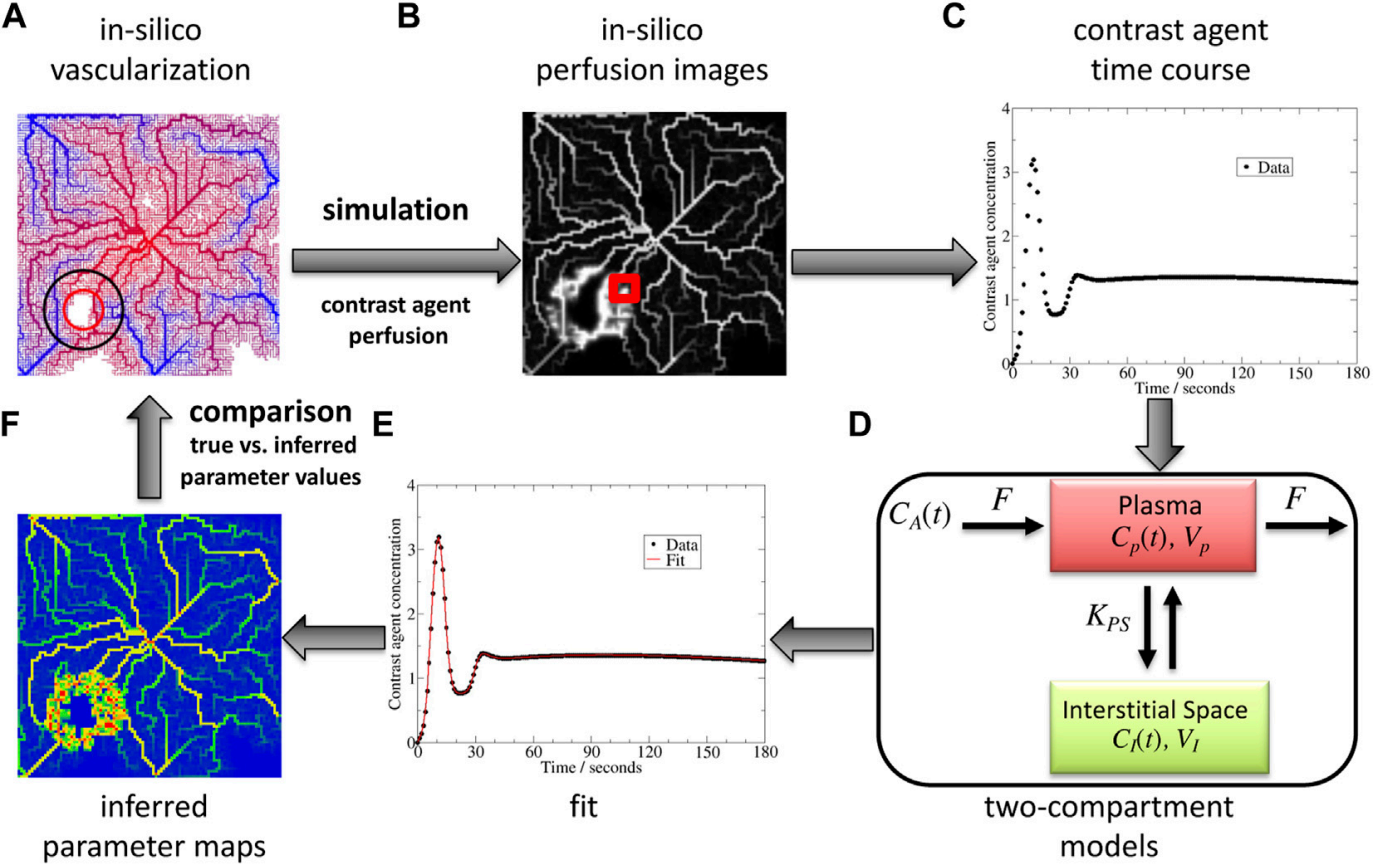
Benchmark concept and its different steps: *in silico* vascular network **(A)**, *in silico* DCE-MRI generation **(B)**, dynamic signal extraction **(C)**, interpretation of the parameters with a tracer kinetics model (here a two-compartment model for extravascular contrast-agent **(D)**) by an inverse procedure **(E)**, and comparison of obtained parameter maps **(F)** with the original ones. *C*_*A*_ represents the input concentration, *F* blood flow, *C*_*P*_ concentration in the vessels, *V*_*P*_ their volume, *K*_*PS*_ the exchange rate with the interstitial space, *C*_*I*_ concentration in the latter, *V*_*I*_ its volume.

**FIGURE 2:**
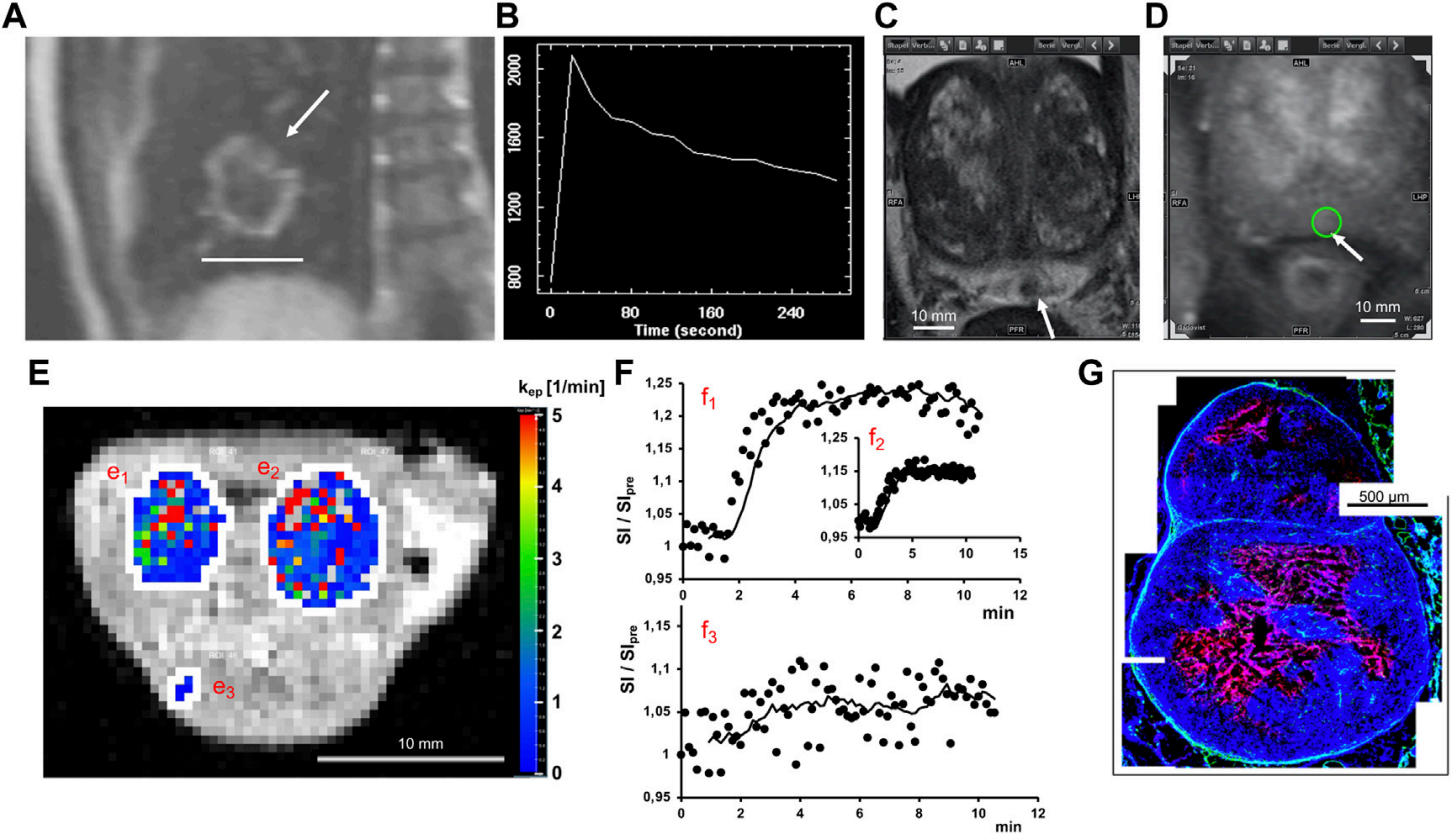
Tissue perfusion characterization. Examples of tumors in human (upper row) and animal model (lower row). **(A)** DCE-MR-Image of Non-small cell lung cancer (NSCLC) tumor showing a characteristic enhanced ring after injection of a contrast agent (arrow: tumor of 4.1 cm (bar)). **(B)** Time course of the DCE-MRI signal intensity measured every 20s in a region of interest for an NSCLC tumor. **(C)** Prostate tumor (3.8 mm) in T2-weighted MRI. **(D)** DCE-MRI image of same tumor (circle). **(E)** DCE-derived parameter map of two Xenograft tumors (e1,e2) and muscle (e3) overlayed on T1-weighted MRI. **(F)** Corresponding signal intensity for tumors (f1,f2) and muscle (f3). **(G)** Histological cross-section of a few mm tumor grown in a Xenograft model showing cell nuclei (blue) surrounding a necrotic area (red).

We here establish a proof of concept for the entire path i.e., a workflow, starting with establishment of a direct model of blood vessel network and function, and simulating in how far two frequently used representatives of TK-models, e.g. the so called Brix and Tofts models, are able to infer information on tissue architecture and function from *in silico* DCE images (Figure 1).Our approach is not exhaustive but guided by Einstein’s principle that “everything should be made as simple as possible, but no simpler” to avoid unnecessary complexity. To limit the number of parameter sets, we focus on those parameter sets, that are capable of reproducing typical DCE-MRI signal patterns (Figure 2). These include the enhancement of a tumor’s border in case its microvascular density is elevated, while the core remains dark indicating necrosis (Figure 2A and movie TumorRimPerf.mov). Also an entire tumor can light up (in particular, if small) (Figure 2E) leading to characteristic fingerprints in the dynamic signal intensity (Figure 2F, f1, f2), which, for example, do not occur in muscle tissue (Figure 2F, f3). Even millimetre tumors are clinically observed (Figures 2C,D), which show a moderate elevated DCE signal (see also Supplementary Appendix SA8) and may display a necrotic core (Figure 2G).The workflow within this work connects a number of building blocks. For each of them, some aspects have already been addressed in published references, which for the sake of clarity we summarize only briefly in the introduction (more extensive information has been depicted in Supplementary Appendix SA2).

The first contribution of this work is a flexible tool for vascular network creation and adaptation, control of features such as regional microvascular density and necrotic core, and computation for flow in this architecture (Figure 1A). The first step is flow modelling in blood vessel networks characterized by hallmarks of cancer vascularization, namely angiogenesis and necrosis (Hanahan and Folkman, 1996; Bergers and Benjamin, 2003; Vaupel, 2004). Stamatelos et al. (2014) were able to reconstruct the microvascular network of tumor Xenografts by a tremendous effort, but for demonstration of our workflow, the required network must be much greater than the tumor itself and its topology under control. Moreover, during the image reconstruction process, network connections may be missed out, which can be avoided if the network is constructed *in silico*. Representing hierarchical arteriovenous networks is however important to study tumor vascularization (Rieger and Welter, 2015). Hence by contrast to previous modelling work that mostly did not take it into account (Drasdo et al., 2010; Perfahl et al., 2011) or that rather focused on the development process (Welter et al., 2008; Welter and Rieger, 2010), we here concentrate on the generated tumor vascularization within a hierarchical network since it can be considered static at the time-scale of dynamic image acquisition. However, we only focus on the microvascular scale as these vessels mostly mediate the exchange of nutrients and contrast agents with the extravascular compartments and as they are representing the angiogenic sites.

The second contribution of this work is in the generation of *in silico* images from the coupled modeling of the contrast-agent in such a tumor vascular network and tissue, which here includes extravasation and diffusion in the interstitial space (Figure 1B). Indeed, given a computed blood flow, the transport of contrast agent or drugs inside these vessels and outside can then be computed. The spread of such compounds has been modelled either only intravascularly or considering a refined model of flow and drug concentration exchange, but not taking into account transport in the hierarchical networks feeding the tumor (Rieger and Welter, 2015). Closer to our work, Mescam et al. (2010) studied a multiscale model of blood flow and contrast-agent spread in liver, to better understand DCE-MRI images in the presence of a tumor. Flow is computed in realistic 3 d networks down to 40 *μm* connected with functional tissue units that do not resolve the microvascular (e.g. capillary) structure. By contrast, the model considered here simulates flow and transport in explicit vascular networks including capillaries, with transport computed inside the networks with emphasis on the essential exchange between the intra-and extravascular spaces. 2 and 3 d vascular networks as well as 1 d vessels are considered. The results show different functional examples and their influence on dynamic perfusion images.

Given a dynamic signal over time (Figure 1C), the next step is to infer some parameters from it, that reflect the underlying tissue (Figures 1E,F). The signal is interpreted at the image voxel level or in a whole region of interest (ROI), typically a region that is thought to be the tumor or a non-tumor zone, by a variety of TK models. None of the ones used in DCE imaging practice take space into account (Sourbron and Buckley, 2013). Semi-quantitative parameters (time to peak, maximum slope, etc.) can be easily extracted but without being specific for the different underlying tissue parameters. This work focuses on TK models that contain parameters surrogate for what is seen in typical tumors such as zones of higher microvascular density or significant leakage (Figure 2): the contrast agent concentration is often described by two interacting compartments, one for blood and one for the extravascular space (Tofts et al., 1999; Lee et al., 2003). It is assumed that the feeding concentration to the blood compartment is the same for all voxels, in some works with a delay-parameter (Lee et al., 2003; Koh et al., 2011; Sourbron and Buckley, 2013). Many TK models fit into the general theoretical framework proposed by Sourbron (2014).The third contribution of this work is in the assessment of the inverse procedure in 1, 2, and 3 d on synthetic DCE imaging data for two common TK models. Overall the goal is to validate the relationship between microscopic information and DCE imaging data *in silico*. Zwick et al. (2010) investigated how parameters of 2 TK models correlate to the tissue input parameters of an effective one-dimensional model of contrast-agent spread inside and out of the microvasculature i.e., without explicitly representing the vascular network and thus the tumor tissue structure in space. The same conceptual idea is pursued here, but starting from a more complex model, where space is explicitly taken into account both in the transport of the contrast agent in branching trees and connected tumor vascularization, and in the possible diffusion of the contrast agent outside the blood vessels, hence different from Zwick et al. (2010) generating spatial perfusion images and parameter maps (Figures 1B,D).

The structure of the paper (*cf.* Table of content before the Reference list) in *Material and Methods* and *Results* follows the different steps of the workflow (see Figure 1); namely functional microvascular network and tumor characteristics, generation of *in silico* dynamic perfusion images and estimation of the corresponding parameters from 2 TK models. This proof of concept of validating the relationship between microscopic data and DCE imaging data and the obtained results are finally discussed. The findings suggest that a more accurate parameter inference may be achieved if the distance of the feeding artery (if it is known) from the ROI is taken into account. The main components of each step of the workflow is explained in Section 2. This section also describes more precisely the novelties. Complementary technical information and results not necessary to understand the main conceptual line of the paper are given in the SI so that the paper is self-contained and the results can be reproduced (see also the *Benchmark code* section; some additional information can be found in ref (Jagiella, 2012)).

## 2 Materials and methods

In the methods section, the spatial vascular architecture and perfusion model are described, followed by the contrast-agent intra- and extravascular transport modeling. Inference procedures are explained based on two common TK models similarly as in Brix et al. (2010).

### 2.1 *In-silico* tumor vascularization

In this subsection we briefly summarize qualitatively how the vasculature is generated with focus on the tumor vasculature (technical details, see Supplementary Appendix SB1). The construction of the vasculature requires and thereby implies the simulation of flow. The structure and remodeling of the microvascular networks closely follow previous works of Goedde and Kurz (2001) or Welter and Rieger (2010). But the purpose here is different: we consider the time scale of diagnosis, not of growth and remodeling. The vascularization is constructed to be directly reflective of different tumor architectures. Hence, novel local rules are proposed for the tumor region.

Vascular networks along with their flow properties (flow-rate, pressure, wall shear-stress) are constructed based on graphs placed on a regular lattice (in 1, 2, 3 d) by placing points into the center of each unit (a cube in 3d, square in 2 d, line segment in 1 d) and linking those with 3 d cylinders according to a certain set of rules specified in Supplementary Appendix SB1.1. Networks are first initialized from arterial and venous tree roots (Supplementary Appendix SB1.2), which become functional, interpenetrating trees connected by capillaries by shear-stress homogenization (Supplementary Appendix SB1.3). Vessel radii are recursively computed from capillaries up to the roots of these hierarchical networks, according to a power-law of coefficient *α* (equation (B.1)).

Inside growing tumors the micro-environment is different than in healthy tissue. In order to create a “tumor-like” vascularization, two regions - a tumor region and a necrotic core - are defined, where the rules and parameters for the homogenization algorithm are different (Supplementary Appendix SB1.3). Due to high proliferation, tumor cells consume much more nutrients than cells in the surrounding healthy tissue. Consequently, an increased production of VEGF in hypo-nourished parts of tumor stimulates vessel sprouting and increases microvascular density (MVD), and a delicate interplay of different growth factors and nutrient availability leads to different tumor vascularization scenarios (Drasdo et al., 2010; Welter and Rieger, 2010). During algorithmic vessel generation the parameter quantifying vessel sprouting (the “vessel sprouting probability” $p_{\mathrm{tumor}}^{\mathrm{spr}}$ ) is increased while another parameter characterizing vessel collapse (the “vessel collapse probability” $p_{\mathrm{tumor}}^{\deg}$ ) is decreased to reproduce a highly angiogenic tumor with PDGF (platelet-derived growth factor) mediated vessel maturation in the *tumor* model (see Table 1). In addition, the higher MVD is mimicked by connecting neighboring capillary ends with *n* parallel vessels, which modifies the relation of vessel radii at branching points and computation of flow along capillaries (Supplementary Appendix SB1.4).

**TABLE 1:** Vascularization parameters specific to healthy, tumorous and necrotic tissue zones. *τ* denotes the wall shear stress, *τ*_min_ and *τ*_max_ minimal and maximal values, respectively (Supplementary Appendix SB1.3).

	Normal tissue	Tumor	Necrotic core
Sprouting Probability	$p_{\mathrm{normal}}^{\mathrm{spr}}=0.5$	$p_{\mathrm{tumor}}^{\mathrm{spr}}=1$	$p_{\mathrm{necrotic}}^{\mathrm{spr}}=0.1$
Degeneration Probability	$p_{\mathrm{normal}}^{\deg}=\frac{\tau_{\max}-\tau }{\tau_{\max}-\tau_{\min}}$	$p_{\mathrm{tumor}}^{\deg}=p_{\mathrm{normal}}^{\deg}/10$	$p_{\mathrm{necrotic}}^{\deg}=p_{\mathrm{normal}}^{\deg}\cdot 10$
Micro-Vessel Density	*n*_*normal*_ = 1	*n*_*tumor*_ = 10	*n*_*necrotic*_ = 10

The plasma volume fraction *ϕ*_*P*_, given by the ratio of volume occupied by vessels to total volume within a tissue region of interest, in tumorous tissue was found increased by one order of magnitude (*ϕ*_*P*_ = 0.04 ± 0.01 in pectoral muscles; *ϕ*_*P*_ = 0.2 ± 0.07 in carcinoma; see (Brix et al., 2004)). In the following, unless otherwise specified, the default value is *n* = 10 for inter-tip-connections inside tumoral regions (see Table 1).

Beyond a certain size, the central parts of the tumor are not nourished sufficiently anymore: a necrotic core appears. It was observed that this necrotic zone is not only lethal to tumor cells, but to endothelial cells and thus to small blood vessels as well (Kiessling et al., 2003; Palmowski et al., 2008a). Thus in this region, the vessel sprouting probability $p_{\mathrm{necrotic}}^{\mathrm{spr}}$ is decreased and the vessel collapse probability $p_{\mathrm{necrotic}}^{\deg}$ increased compared to healthy tissue (see Table 1).

### 2.2 *In-silico* contrast-enhanced perfusion images

The vascular network structures are then used to simulate the transport of contrast-agents through vascularized tissues and create *in silico* perfusion images.

#### 2.2.1 Contrast-agent modeling

Multi-phase equations model the macroscopic transport of the contrast agent in the tissue namely both inside the vascular network and the interstitial space. This model fits into the class of multiphase reaction-advection-diffusion models that for tracer kinetic models have been suggested to be generated by combining key process modules, namely, convection, diffusion, leakage, absorption, decay and sources (Sourbron, 2014). See Supplementary Appendix SB2.1 for its link to microscopic model and interphase exchanges.

The mass concentration of contrast-agent (CA) inside the plasma follows

$$\begin{array}{cc} \frac{\partial }{\partial t}\left(\phi_{P}c_{P}\right)+\nabla \cdot \left(\phi_{P}c_{P}v\right) & =-k_{PS}\left(c_{P}-c_{I}\right)\text{ in }\Omega ,\\ {\left.c_{P}\right|}_{\partial \Omega_{in}} & =c_{A}\left(t\right), \end{array}$$ (1)

where *c*_*A*_ is the arterial (or more generally, vascular) input function (AIF), the blood plasma concentration in the arterial root nodes *∂Ω*_*in*_. *c*_*P*_ = *n*_*P*_/*V*_*P*_ is the macroscopic (local averaged microscopic) plasma CA concentration, and *c*_*I*_ = *n*_*I*_/*V*_*I*_ is the macroscopic (local averaged microscopic) interstitial CA concentration, whereby *n*_*P*_, *n*_*I*_ are the number of CA molecules in phases P and I, *V*_*P*_ and *V*_*I*_ the volumes of phases P and I in the tissue volume *V* = *V*_*P*_ + *V*_*I*_. **v** is the macroscopic blood velocity, and *k*_*PS*_ the volumetric macroscopic membrane exchange rate, both defined later. Parker et al. (2006) proposed an AIF functional form derived from patient data (see Supplementary Appendix SB.2.2, Supplementary Table SB4).

Assuming no advection but only diffusion in the interstitial space as a consequence of cells and extracellular matrix, and no-flux conditions at the tissue border, the CA mass concentration in the interstitial space follows

$$\begin{array}{cc} \frac{\partial }{\partial t}\left(\phi_{I}c_{I}\right) & =\nabla \cdot \left(\phi_{I}D_{I}\nabla c_{I}\right)+k_{PS}\left(c_{P}-c_{I}\right)\text{ in }\Omega ,\\ {\left.\frac{\partial c_{I}}{\partial n}\right|}_{\partial \Omega } & =0 \end{array}$$ (2)

where *ϕ*_*I*_ *D*_*I*_ is the effective diffusion coefficient due to the fact that the molecules are not diffusing freely among the whole volume, but are limited by the cells and vessels borders. *D*_*I*_ is taken as 10^3^ *μm* ^2^/*s* (Lemke et al., 2009) while *k*_*PS*_ will be defined later. For an in-depth derivation of the macroscopic equations from homogenization theory, see (Penta et al., 2015).

#### 2.2.2 Generation of parameter maps and *in silico* images

Eqs 1, 2 are then discretized and solved numerically for each voxel *i* (see Supplementary Appendix SB2.3). The flux *f*_*i*,*j*_ from node *i* to node *j* relates to the velocity *v*_*i*,*j*_ as $f_{i,j}=v_{i,j}\pi r_{i,j}^{2}$ . The nodes are placed in the middle of each voxel (volume element on the regular lattice), *i* serves at the same time as a lattice identifier (Supplementary Appendix SB1.1). Parameter maps can then be defined for all voxels of an *in silico* image. Given the vascular graph G (defined in Supplementary Appendix SB1.1) gives for each voxel i its connected voxels j, the vascular volume fraction for voxel *i* of volume *V*_*i*_ is given by

$$\phi_{P,i}=\frac{V_{P,i}}{V_{i}}=\frac{\pi }{2l^{2}}\underset{\mathrm{j\in}}{\sum }r_{i,j}^{2}.$$ (3)

*V*_*P*,*i*_ being the voxel vascular volume, and the extra-vascular volume fraction is

$$\phi_{I,i}=1-\phi_{P,i}.$$ (4)

The associated flow rate *F*_*i*_ is the sum of all entering fluxes:

$$F_{i}=\underset{j\in \text{connected neighbors}}{\sum }f_{j,i}{|}_{+}.$$ (5)

The volumetric exchange rate *k*_*PS*,*i*_ in voxel *i* relates to the exchange rate *K*_*PS*,*i*_ as *k*_*PS*,*i*_ = *K*_*PS*,*i*_/*V*_*i*_ in a voxel *i*. This exchange rate between the plasma and the interstitial space can be written as the product of the surface of the blood vessel walls *S*_*i*,*j*_, and the permeability coefficient, *P*_*i*,*j*_:

$$K_{PS,i}=\underset{j\in \text{connected neighbors}}{\sum }\frac{P_{i,j}S_{i,j}}{2}=\underset{j\in \text{connected neighbors}}{\sum }P_{i,j}\pi lr_{i,j}$$ (6)

By identification with the microscopic scale, *P*_*i*,*j*_ represents $D_{i,j}^{m}/H_{i,j}^{m}$ . Note, that half of the connecting vessel between two nodes *i*, *j* is associated with voxel *i*, the other with voxel *j*, which explains the division by a factor 2. In the rest of the paper, the permeability coefficient *P*_*i*,*j*_ is a homogeneous constant noted *P* that can vary between healthy and tumor regions. Finally, the total concentration in a voxel *i* is then naturally defined as:

$$c_{i}\left(t\right)=\phi_{Pi}c_{Pi}\left(t\right)+\phi_{Ii}c_{Ii}\left(t\right)$$ (7)

In case the ROI is a volume that contains several voxels, the *in silico* generated measurement is the total concentration averaged over all voxels of the ROI. The associated parameters are:

$$\begin{array}{cc} \phi_{P,ROI} & =\underset{i\in ROI}{\sum }\phi_{P,i}\\ F_{\mathrm{ROI}} & =\underset{i\in ROI}{\sum }\underset{j\in \left\{\text{connected neighbors of i}\right\}\cap j\notin ROI}{\sum }f_{j,i}{|}_{+}\\ K_{PS,ROI} & =\underset{i\in ROI}{\sum }K_{PS,i} \end{array}$$ (8)

Here it was used that all voxels are by construction of the same size, and *K*_*PS*_ is an extensive quantity.Finally, the total concentration relates differently to the signal intensity depending on the image modality (Brix et al., 1991; Hoffmann et al., 1995; Brix et al., 2004). Without loss of generality, it is here directly taken as the surrogate for the *in silico* image signal over time in each voxel.

#### 2.2.3 Direct model parameters

The direct model parameters are not always known, and hence to choose representative values is a challenge. The network is constructed to reflect the increase from healthy to tumor tissue of plasma volume fraction (section 2.1). The healthy value is taken from muscle and can be changed. Tumor values are more variable (e.g. (Brix et al., 2004) reporting ranges of 0.1–0.4), and are extensively varied here (Supplementary Appendix SC3). A baseline permeability of *p* = 0.1 *μm*/*s* is used everywhere (in healthy and tumor zones). This value is inferred from (Brix et al., 2004; Brix et al., 2009) who found close *K*_*PS*_/(*ϕ*_p_*V) values for both muscle and carcinoma tissues, by estimating *S*/*V* and *S*/(*ϕ*_p_*V) from this paper’s networks and assuming a tissue density of 1*g*.*mL* ^−1^. *p* = 0.1 *μm*/*s* was also reported for caco-2 (adenocarcinoma) and *p* = 0.35 *μm*/*s* MDCK (kidney) cells for paracetamol (Irvine et al., 1999), which has a diffusion coefficient of approximately 650 *μm* ^2^/*s* Ribeiro et al. (2012) close to what is chosen in this work. In Mescam et al. (2010); Zwick et al. (2010), *K*_*PS*_/*V* is varied from 0, 0.01, 0.1 and up to 1 min^−1^. Taking all this information into account motivated to vary *P* from 0, 0.01, 0.1–1 *μm*/*s* in most cases. These papers focused on gadolinium contrast agents, but permeability can be further varied.

### 2.3 Estimation of functional parameters

DCE images give a value of the total CA concentration in every region of interest (ROI) for each acquisition time. The exact structure of the vascularization in each ROI and the interconnection between ROIs are completely unknown. In DCE imaging, simplified non-spatial models representing in each ROI separately the circulation of CA and its exchange with the rest of the tissue are fitted in an inverse procedure to recover perfusion and permeability information from the imaging dynamic curves.

#### 2.3.1 Tracer kinetic models

In DCE imaging, a number of two-compartment models (Brix et al., 1991; Tofts and Kermode, 1991; Brix et al., 1999; Tofts et al., 1999; Sourbron, 2014) are the basis for quantification of regional blood flow, vessel permeability, and relative compartmental volumes within an ROI. In the following we will focus on two most common models proposed by Brix et al. (1999) and Tofts et al. (1999).

##### 2.3.1.1 The Brix II model

This model, sometimes in the literature referred to as 2CXM (Sourbron and Buckley, 2013), takes into account in each ROI the blood (or plasma) and interstitial (extravascular) compartments (see Supplementary Figure SC13) of respective relative volumes *ϕ*_*P*_ and *ϕ*_*I*_ in ROI volume *V*, coupled by the intra-vascular-extra-vascular exchange rate *K*_*PS*_.

Each ROI blood compartment is fed directly from the AIF, with blood flow rate *F* going in and out of the compartment. No further transport or diffusion are taken into account. The corresponding equations are thus

$$\frac{d\left(\phi_{P}C_{P}\right)}{dt}=\frac{F}{V}\left(C_{A}-C_{P}\right)-\frac{K_{PS}}{V}\left(C_{P}-C_{I}\right)$$ (9)

$$\frac{d\left(\phi_{I}C_{I}\right)}{dt}=\frac{K_{PS}}{V}\left(C_{P}-C_{I}\right)$$ (10)

The total concentration of contrast agent in the tissue is thus assumed to be defined similarly to Equation 7. These equations are solved numerically with an implicit Euler scheme.

##### 2.3.1.2 The Extended Tofts model with and without delay

Tofts et al. (1999) proposed another two-compartment model (sometimes called *Generalized Kinetic model*), first neglecting the blood contribution assuming a negligible blood volume.

$$\frac{d\left(\phi_{I}C_{I}\right)}{dt}=K_{\mathrm{trans}}\left(C_{P}-C_{I}\right)$$ (11)

In the extended Tofts version, the blood pool contribution is added (Sourbron and Buckley, 2011). The total tissue concentration is then:

$$C\left(t\right)=\phi_{P}C_{P}\left(t\right)+K_{\mathrm{trans}}\int_{-\infty }^{t}C_{P}\left(t^{\prime }\right)e^{-K_{\mathrm{trans}}/\phi_{I}\cdot \left(t-t^{\prime }\right)}dt^{\prime }.$$ (12)

*ϕ*_*P*_ and *ϕ*_*I*_ being the plasma and interstitial compartment relative volumes respectively. The second term on the right hand side of Eq 12 denotes the solution of Eq 11, which emerges if the plasma volume is neglected (*ϕ*_*p*_ = 0). *C*(*t*) is here explicitly given as a function of the CA plasma concentration *C*_*P*_(*t*) assumed to be the AIF, and of a transfer constant *K*_*trans*_ × *V* whose meaning depends on the balance between the aforementioned flow *F* and permeability *K*_*PS*_ (Tofts et al., 1999). As the AIF *C*_*A*_(*t*) might be measured in an area far away from the ROI, this assumption may not hold in most of the cases. Thus, a parameter *t*_0_ can be added (Koh et al., 2011; Sourbron and Buckley, 2013), accounting for the time delay between the measured signal in the feeding artery and the vessels in the ROI. Eq. 12 becomes

$$C\left(t\right)=\phi_{P}C_{A}\left(t-t_{0}\right)+K_{\mathrm{trans}}\int_{-\infty }^{t}C_{A}\left(t^{\prime }-t_{0}\right)e^{-K_{\mathrm{trans}}/\phi_{I}\cdot \left(t-t^{\prime }\right)}dt^{\prime }.$$ (13)

#### 2.3.2 Parameter inference

The feeding concentrations *C*_*A*_(*t*) are assumed to be the same as the previously defined AIF *c*_*A*_(*t*). In practice, the dynamic signal for a given ROI is acquired with a certain temporal resolution Δ*t*, for *N*_*t*_ time steps of index *n*. This ROI-CA concentration at each time step *c* (*n* ⋅Δ*t*) is compared to the TK solution *C* (*n* ⋅Δ*t*) for this ROI with the *L*_2_ norm *S* covering the entire acquisition time:

$$S\left(\beta \right)=\overset{N_{t}}{\underset{n=1}{\sum }}{\left(c\left(n\cdot \Delta t\right)-C\left(n\cdot \Delta t,\beta \right)\right)}^{2}$$ (14)

Note that *S*(*β*) corresponds to the quadratic error. The same formula directly permits quantifying the goodness of the agreement between the ROI-CA and the TK solution of both Brix II and DE-Tofts model, as both have the same number of parameters (3, see below). To get the best fitting set of parameters *β*, *S* is minimized with the Levenberg-Marquardt algorithm with 100 iterations. For both tracer kinetics models, as a first approximation Eq. 4 is added as a constraint, since this is how the *in silico* dynamic signal is generated. For the Brix II model, *β* = {*ϕ*_*P*_, *F*, *K*_*PS*_} whereas for the Delay Extended Tofts Model (named thereafter *DE-Tofts model*) *β* = {*ϕ*_*P*_, *K*_*trans*_ × *V*, *t*_0_}.

An interesting information may be obtained by directly comparing the inferred parameters of the Brix II and the DE-Tofts model, which is straightforward for *ϕ*_*P*_ and relatively straightforward for *K*_*PS*_ = *K*_*trans*_ × *V*, but there is no simple equivalence in the DE-Tofts model, that corresponds to the flow parameter *F* in Brix II. As the information of the volume flow rate F in the Brix II model has to be related to a parameter combination of the three Tofts model parameters, a first approach is by dimensional analysis, leading to the possible definition $F=\frac{\phi_{P}\;V}{t_{0}}$ . However, as will be explained in the Results (Section 3.2.1), this definition needs to be modified. This modification for DE-Tofts gives a rational to do the same for the Brix II flow. When necessary to make a distinction between the flow F from the original map (Eq. 5) and the recovered one from the inference procedure, the latter is then labeled *F*_*recov*_.

## 3 Results

First the results of the generation of *in silico* vascularizations and their validation against known architectural and functional properties are summarized. Then, the *in silico* dynamic contrast-enhanced images, the last step of the direct problem, are presented. The final part focuses on the results of the kinetics models assessment, i.e. the comparison of the parameters estimated with these models, against the original parameters from the *in silico* model. This leads to the proposal of a new interpretation of the estimated parameters to improve the perfusion assessment from the dynamic images.

Note that the 2 d models are 60 *μm* thick tissue slices, i.e. have a thickness of 60 *μm* ≪ extension in (x,y)-plane. The vascular graph itself lies on a 2 d plane with vessels having a certain cylinder volume, thus forming a connected network of a certain plasma volume as described in the Methods. The 3 d model is based on a vessel graph that extends in each of *d* = 3 dimensions. In all dimensions, each voxel has thus by default a size of (60 *μm*)^3^.

### 3.1 From vessel architecture to *in silico* dynamic contrast-enhanced images

#### 3.1.1 Functional vascularization

Firstly, we verified that the vascular network generation algorithm (see Section 2.1) generates vessel networks that reproduce known morphological and functional features. Initially, the arterial and venous trees stay well separated from each other (Supplementary Figure SC11). Large parts of the vascular networks have low to almost no flow, and, consequently, are not functional. The construction algorithm is iterative; with the number of iterations, inter-tree-connections increase and with them the overall flow, resulting in interpenetrating hierarchical arterio-venous networks with a homogeneous wall shear stress in both the 2d and 3 d models (see Supplementary Appendix SC1).

**FIGURE 3:**
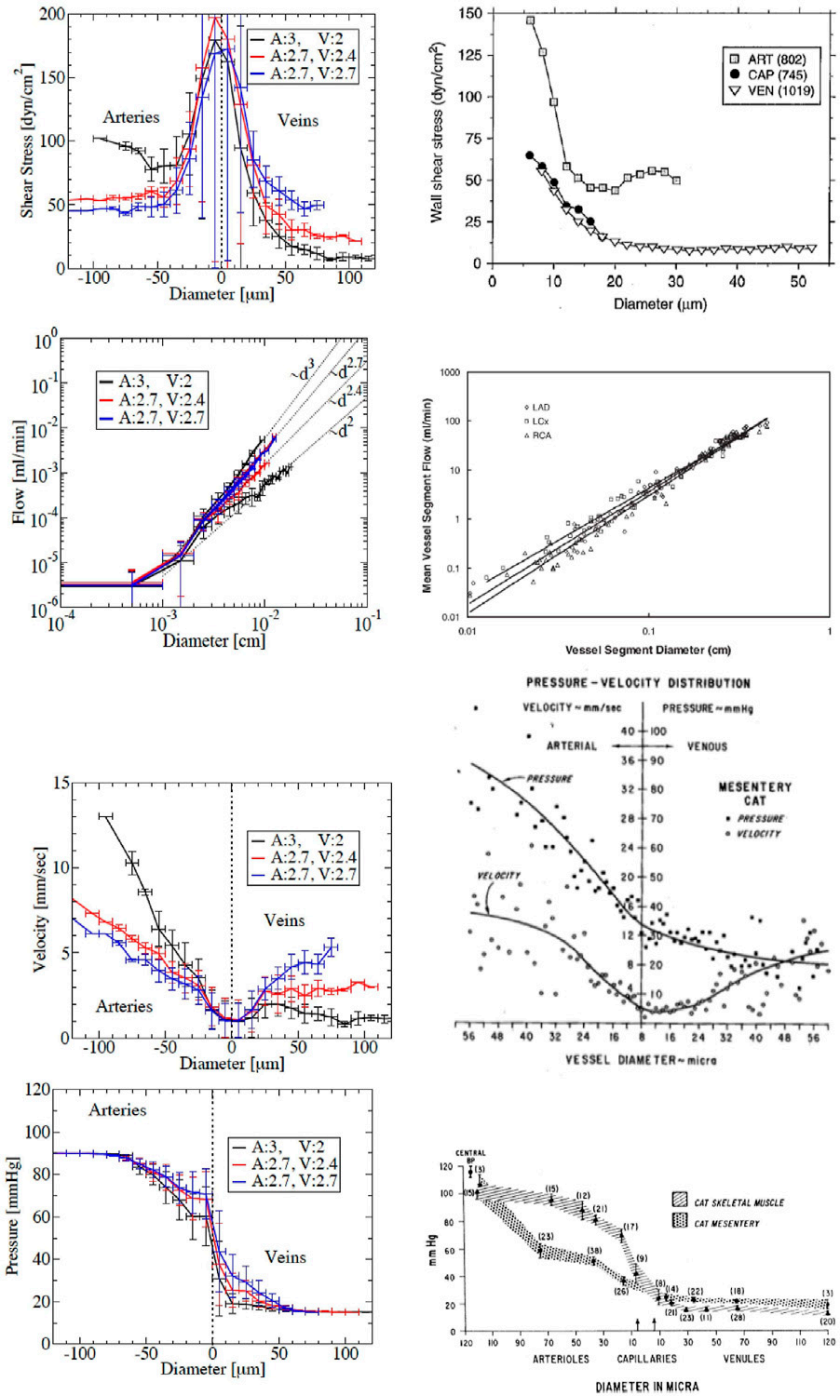
Comparison with experimental measurements. Left: simulation results for three different arterial-venous trees with pairs of exponent *α* (denoted by A for the arterial, V for the venous tree), with the same applied inlet and outlet pressures: in black the pair (A,V)=(3,2), in red the pair (2.7,2.4) and in blue the even pair (2.7,2.7). For shear stress, velocity and pressure, arteries are shown on the negative diameter side, while veins are on the positive diameter side. For flow their trees are plotted together to demonstrate that flow in arteries and veins follow the same power laws as the corresponding diameter (*d* ^*α*^), and can be identified by their exponent. Right: literature data. The different rows show wss (from Fung (2013)), flow (from Huo and Kassab (2007)), velocity (from Pries and Secomb (2008)) and pressure (from Fronek and Zweifach (1974)), *versus* diameter.

Importantly, the model results actually compare well with measured data on wall shear stress, mean vessel segment flow, pressure-velocity distribution and pressure, in terms of order of magnitudes and changes with vessel radius (Figure 3, right column).

The precise relations depend on the exponents *α* ^*art*^ and *α* ^*ven*^ of the power-law (equation B.1). In several statistics of the obtained micro-circulation properties, we observe differences between vascular trees (e.g. pressure, see Figure 3) and asymmetries between venous and arterial vessels (e.g. wall shear stress and blood velocity, see Figure 3). Three different *in silico* vascularizations in 2d, with varying exponents *α* ^*art*^ and *α* ^*ven*^ are considered. The higher the exponent *α*, the higher the flow for a given applied pressure drop (Figure 3, second row) indicating a drop of flow resistance. This results from two competing effects that can also explain the differences of decay between flow rate, velocity and wall shear stress with respect to diameter when increasing the exponent (see Supplementary Appendix SC2). The best fit of the asymmetric properties between model and data leads to choose for the rest of the article different default values for arteries, *α* ^*art*^ = 3 and for veins, *α* ^*ven*^ = 2.7 (Figure 3).

#### 3.1.2 Vascularized tumor cases

After having generated normal vascularizations, we now study vascularized tumor cases for various MVDs reflected in the parameter value of *n* defined in equation B.5) (Supplementary Figure SC12). Above a certain MVD volume fraction *ϕ*_*P*_ becomes locally sufficiently different from its values in neighboring regions (second row in Supplementary Figure SC12). The higher volume fraction leads to a higher flow, also well detectable already at smaller *n* (last row in Supplementary Figure SC12). In the necrotic core (Supplementary Figure SC12 last column), the model induces the destruction of the small vessels in it, but the larger vessels remain inside it. In the tumor periphery, there is an increased *ϕ*_*P*_. The latter corresponds to a characteristic observation in patient tumors (Figure 2A). This structure of larger vessels remaining inside tumors with low vessel density in the center and high density at the periphery is typically observed in multi-modality imaging in Xenograft experiments (Kiessling et al., 2004b,a). The later also reports dilated vessels feeding the tumor as emerges in the model here as well.

#### 3.1.3 *In-silico* dynamic contrast-enhanced images

**FIGURE 4:**
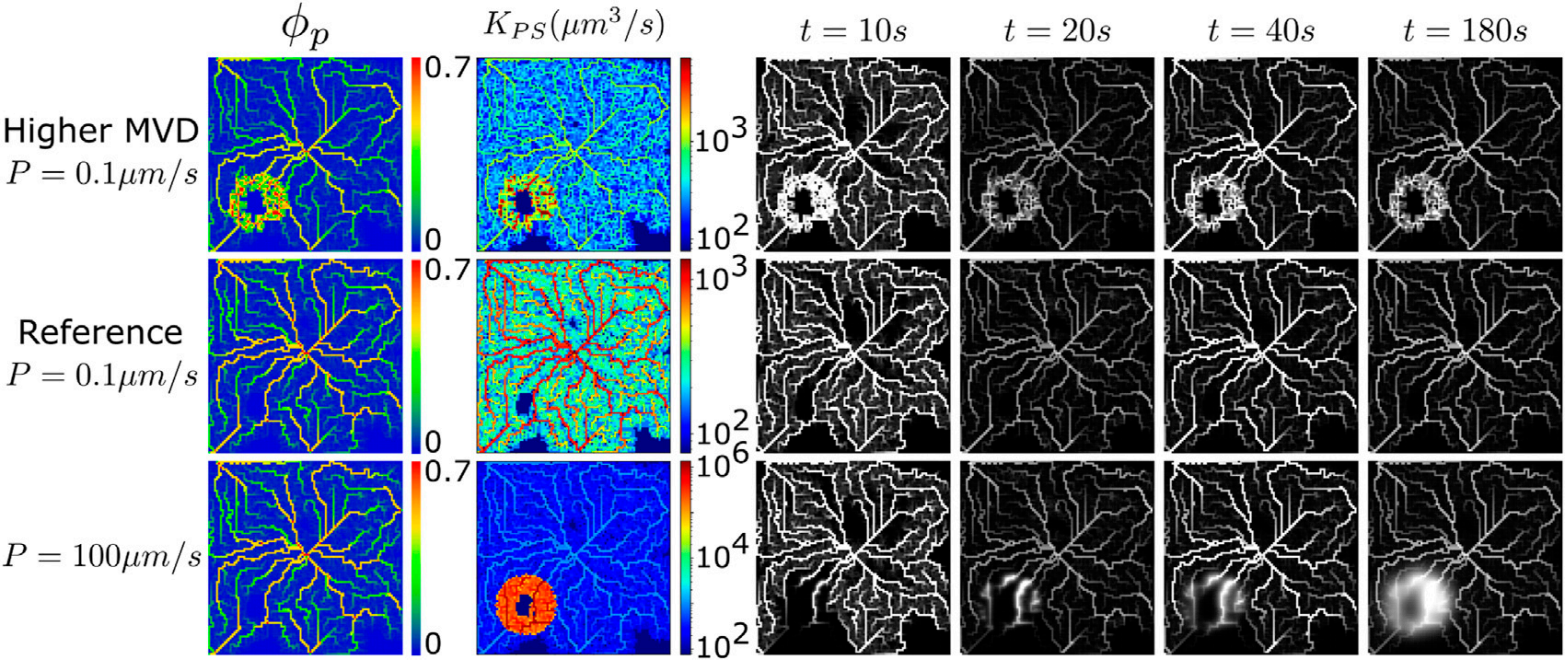
Increased MVD and/or permeability. Increased MVD in the tumor region (first row) by comparison to the reference simulation (second row), with permeability *p* = 0.1 *μm*/*s* for these two simulations. Increased permeability in the tumor region (*p* = 100 *μm*/*s*, last row) by contrast to the reference simulation, with normal MVD for these two simulations. Plasma volume fraction (first column, *ϕ*_*p*_), exchange rate *K*_*PS*_ (second column, in *μm* ^3^/*s*) and *in silico* DCE images at four different times (remaining columns). *K*_*PS*_ in *μm* ^3^/*s*. Voxels of (60 *μm*)^3^, domain:100 × 100 voxels. Movies: see list in Supplementary Appendix SC3.

Based on microcirculation reflective of the normal and tumor regions, *in silico* DCE images (quantifying *c*(*t*) as defined in Eq. 7), illustrate the kinetics specific to each component (Figure 4; and movies for further varied parameters in Supplementary Appendix SC3). The reference case leads to a quite homogeneous vascular volume fraction and hence capillary-tissue exchange rate *K*_*PS*_. The larger vessels are visible due to their larger values. The corresponding *in silico* overall image intensities follow in their time course the kinetics of the AIF, with the peak of the first pass, then a large decrease, then a second peak followed by a slower decay. Compared to the average image intensity, the larger vessels are highlighted, and the zones of capillary-tissue exchange appear as a haze. By contrast, as soon as MVD is locally increased (*n* = 30) in the tumor region, this increases the local capillary-exchange rate. On top of the previously described dynamics, the tumor outer-rim always appears highlighted. Modelling the tumor by an increased permeability (*p* = 100 instead of 0.1 *μm*/*s*) only also leads to a local capillary-tissue exchange rate increase. However, the *in silico* DCE-images are different (Figure 4 bottom row): the tumor is highlighted much later, with a diffuse glow around the entire tumor zone. The explanation is that in case of elevated MVD the simulated signal intensity reflects directly the MVD and hence, follows the kinetics of tumor perfusion, dictated by the AIF, while in case of an elevated permeability, the marker leaks into the extra-vascular space and returns as soon as the vessel marker concentration drops below the extra-vascular marker concentration, which generates the delay.

These results correspond to well-known tumor enhancement differences. For example, hepatocellular carcinomas, neuroendocrine tumors, renal cell carcinomas, and squamous cell carcinomas have strong arterial feeding and high MVD and thus, are clinically characterized by their early enhancement after contrast agent administration in CT and MRI scans. The other extreme are low to moderately differentiated gliomas where the disruption of the blood brain barrier leads to enhanced vascular permeability. These tumors are best seen in late enhancement phases after contrast agent injection, when the contrast agent had sufficient time to slowly distribute in the extravascular tumor space. These special vascular features are of high importance for diagnosing and differentiating many tumors and have strongly influenced the clinical guidelines on the performance of CT and MRI examinations.

Hence, the different components of what defines a vascularised tumor lead to different dynamical features of the DCE-images. It is thus natural to extract quantitative differences in parameters based on the dynamics differences seen in DCE images. This is the focus of the next section.

### 3.2 Inference results

The problem of inferring model parameters from a dynamic intensity in a region of interest is called an *inverse problem*, or *inverse procedure*. This procedure requires to have a measurement (here the concentration over time) and to select a model to interpret this measurement. *In-vivo* the exact mathematical model that represents this dynamics is not known, and in practice simplified models are selected. Here we generate *in silico* images with a known model, and perform the inverse problem with simplified models (Brix II and DE-Tofts models) to better understand their relevance.

#### 3.2.1 Intravascular agents

**FIGURE 5:**
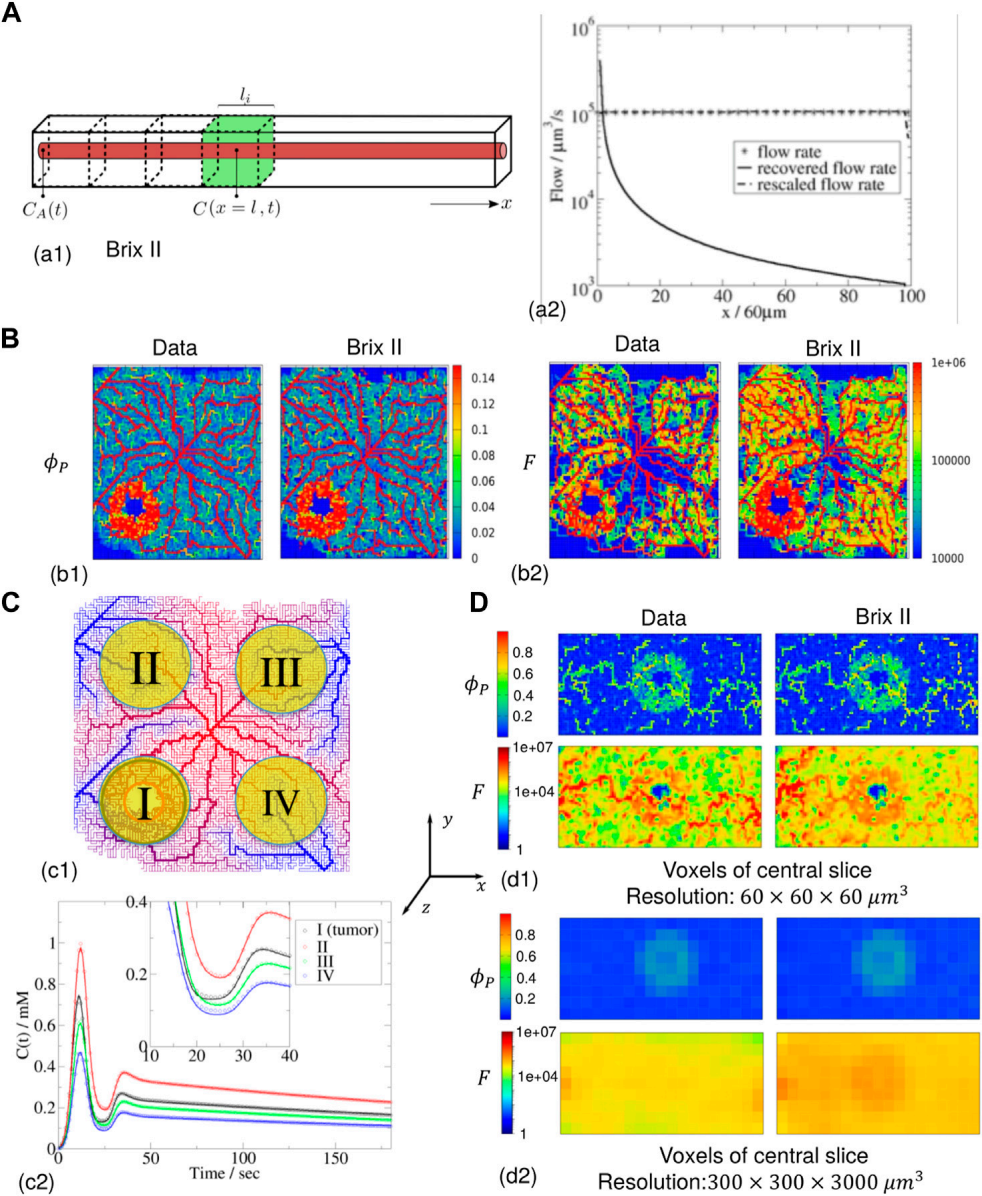
Inference for zero permeability **(A)** 1 d case geometry (green voxel of (60 *μm*)^3^) and flow parameter along the 100-voxel domain (data, recovered and rescaled). **(B)** 2 d case of 100 × 100 voxels with the data (left column) and the Brix II recovered maps (right column) (DE-Tofts: see **(C)** 15) for (60 *μm*)^3^ voxels. The feeding artery is in the center of the image (c1): vascularization topology (from high (red) to low (blue) pressure) and four different regions of interest (ROI size = 709 voxels, ≈2 mm diameter). I contains the tumor necrotic zone (red circle) inside the tumor rim (green circle). Feeding artery in the center of the image (c2): the fit (continuous curves) of the measured contrast-agent concentrations over time (circles) for the four zones by the Brix II model. **(D)** Parameter inference from 3 d data (6*mm* × 3*mm* × 3 *mm*). The feeding artery is on the left of the images (central slices in *z*-direction) (d1) *ϕ*_*p*_ (1rst row) and flow (second row) for a (60 *μm*)^3^ resolution; (d2) *ϕ*_*p*_ (1rst row) and flow (second row) for a 300 × 300 × 3000* μm* ^*3*^ resolution. Columns: data, Brix II **(A–D)** *F* is in *μm* ^3^/*s*.

As a reference case we study intravascular agents in one, two and three dimensions (= *d*) (Figure 5). The advantage of performing *in silico* simulations is the opportunity of such a strategy starting with a great simplification, and then step-wise studying more complex cases gives the opportunity of gaining a step-wise understanding.

##### 3.2.1.1 The one dimensional case

In a first step we study the inverse procedure based on Brix II and Tofts models in the simplemost case, which is a one-dimensional straight piece of tissue, as for this case the concentration over time can be analytically calculated. Despite this case looks extremely simplified, it may be compared to the case where the ROI is small compared to the distance between the feeding point and the ROI, in which case the Euclidean distance between feeding point and ROI may be of the order of the distance that the blood has to travel between them. The tissue piece is assumed to be a rectangular cuboid of length of 6 *mm* and cross-sectional area of L*L = 60 μm*60*μm*, containing a straight cylindrical capillary inside of constant radius *r* = 4 *μm* (thus *ϕ*_*P*_ ≈ 0.014) along the long axis (see Supplementary Figure SC13). An intravascular agent enters at the inlet (at *x* = 0) with the known concentration *C*_*A*_(*t*) taken equal to the AIF. In that case, the plasma concentration *c*_*P*_ is obtained from Eq. (7) by dropping the vessel leakage term to

$$c_{P}\left(x,t\right)=C_{A}\left(t-x/v\right)=C_{A}\left(t-x\pi r^{2}/F\right)=C_{A}\left(t-x\phi_{P}L^{2}/F\right),$$ (15)

representing “travelling wave” i.e., a concentration profile that performs a rigid movement with a certain velocity *v* = *F*/(*ϕ*_*P*_ *L* ^2^) (*F* is the volume flow rate, *ϕ*_*P*_ the plasma volume fraction).

The common case is when the ROI does not contain the feeding point of the contrast-agent (Figure 5A1, Supplementary Figure SC13C) (the case where the ROI contains the feeding point is considered in Supplementary Appendix SC4, Supplementary Figure SC13A, B). Consider as a measurement a fixed volume *V*_*i*_ = *L* ^2^*l_*i*_ = (60 *μm*)^3^ but which center is at varying distance *x* from the feeding point (green volume in Figure 5A1). Considering conservation of mass, equation C.1 becomes $\frac{d\bar{c}}{dt}=\frac{d\left(\phi_{P}{\bar{c}}_{P}\right)}{dt}=\frac{F}{V_{i}}\left(C_{A}\left(t-\left(x-l_{i}/2\right)/v\right)-c_{P}\left(x+l_{i}/2,t\right)\right)=\frac{F}{V_{i}}\left(C_{A}\left(t-\left(x-l_{i}/2\right)/v\right)-C_{A}\left(t-\left(x+l_{i}/2\right)/v\right)\right)$ . In this case the ratio of volume and flow, or equivalently *l*_*i*_/*v*, are small relative to the characteristic duration of the signal represented by the time width of the CA concentration peak (Figure 1), *l*_*i*_/*v* < time-width of CA peak, or equivalently *F*/(*ϕ*_*P*_ *V*_*i*_) > 1/time-width of CA peak. The fact of replacing the outlet concentration at position *x*_*i*_ + *l*_*i*_/2 by the average concentration in the inverse Brix II model (Eq. (9)) is a good approximation. The issue is the inlet flux: with increasing *x*, taking *C*_*A*_(*t*) instead of *C*_*A*_ (*t* − (*x* − *l*_*i*_/2)/*v*) is becoming decreasingly accurate (see Supplementary Appendix SC4.1). Thus Eq. (9) under-predicts more and more the flow, as the estimated flow *F*_*recov*_ ≈ *F*/*N*_*i*_, where *N*_*i*_ = *x*/*l*_*i*_ (Figure 5A2) i.e., *F*_*recov*_ *x* is a constant that depends on the chosen size of the ROI, *l*_*i*_. This is validated by the fact that rescaling the recovered flow *F*_*recov*_ by multiplying it by *N*_*i*_, one recovers the exact flow (Figure 5A2, Supplementary Figure SC13C).

In a next step, we consider the DE-Tofts model for the same configuration (Supplementary Figure SC13D). In this model, we have *apriori* defined the estimated flow as *F*_*recov*_ = *ϕ*_*P*_ *V*/*t*_0_. In our simple case, Eq. 13 written for an intravascular agent reduces to *ϕ*_*P*_ *C*_*A*_(*t* − *t*_0_). On the other hand, the analytical solution (Eq. 15) at *x* identifies *t*_0_ = *xϕ*_*P*_ *L* ^2^/*F*. So for an ROI small enough, in the sense described above of *F*/(*ϕ*_*P*_ *V*) > 1/time-width of CA peak, the concentration is sufficiently homogeneous in the ROI and *l* ≈ *N*_i_*l_*i*_. Hence when taking this ROI concentration for the parameter inference, one recovers *t*_0_, and thus *F*_*recov*_ = *ϕ*_*P*_ *V*/*t*_0_. But as in fact, *t*_0_ ≈ *N*_*i*_ *ϕ*_*P*_ *V*/*F*, the recovered flow *F*_*recov*_ ≈ *F*/*N*_*i*_. Thus, here too it needs to be rescaled by the voxel-distance to the feeding point to recover the original flow *F*.

*Sensitivity to noise*: As described in Supplementary Appendix SC4.2, a measurement error or random noise in the tissue measurement affects the inference of the flow more than the inference of the plasma volume fraction. The latter is always estimated with an accuracy of ∼±20% even for noise up to 20%.

##### 3.2.1.2 Two dimensional case

In a next step the case of intravascular agent propagation through a vasculature which contains a vascularised tumor is considered (Figure 5B). The *in silico* contrast-agent images are generated as in section 3.1 (displayed in Supplementary Figures SC11, 12), with blood and the contrast-agent entering through an artery in the middle of the tissue, the contrast-agent feeding temporal profile following the typical AIF of equation Supplementary Appendix SB2.2. The corresponding plasma volume fraction (Figure 5B1) and flow (Figure 5B2) range from 0 to 0.14 and 0–10^6^ *μm* ^3^/*s* respectively, while *P* = 0 *μm*/*s*. The measured concentration in each voxel, computed as the solution to Eq. (7), is the input to the inverse procedure.

Without assuming that the agent is intravascular, the estimated parameters are thus the plasma volume fraction, the plasma-tissue exchange coefficient and the flow. For Brix II, the estimated plasma volume fraction map is visually identical to the initial one (Figure 5B1 right vs. Figure 5B1 left): the main vasculature structure, and the vascularized tumor zone are well detected. Finally, the estimated flow map (Figure 5B2 right), shows the microvasculature structure and the vascularised tumor zone. Due to the rescaling of the flow with the Euclidean distance of the voxel to that feeding point, the flow maps are close to the initial data (Figure 5B2 left). Without the rescaling, the flow going away from the central feeding point is rapidly underestimated, similar to the 1d case (Supplementary Figure SC15). The DE-Tofts model gives very similar results to the Brix II model (Supplementary Figures SC15 right). While in regions where the plasma volume fraction is non-zero, the exchange coefficient is negligibly small as expected, in regions of zero plasma volume (and very low flow), as for example in the necrotic region of the tumor, the exchange coefficient deviates from zero (Supplementary Figures SC15 third row).

##### 3.2.1.3 ROIs of multiple voxels

Next the signal considered for the inverse problem is for a region of interest (ROI) larger than one voxel, as this is often the case in practice (Figure 5C). First, a 2 d vascularised tumor case is modelled with the transport of an intravascular contrast agent (Figure 5C1). The measurements from which parameters are estimated are the spatial average concentrations in four different ROIs, three in the normal zone (II, III, IV) and one containing a tumor (I). Each region contains 709 voxels, thus are all of diameter around 2 *mm* (typically a few in-plane voxels on DCE-MRI images). The concentrations for the four ROIs are similar to the AIF, although with a peak scaling from 0.46 to 1 *mM* and some time-dispersion due to ROI averaging (see zoom picture in Figure 5C2). For both Brix II and DE-Tofts, the measurement curves are well reproduced after parameter estimation, for all four regions, with small deviations between the two first passes as seen in the zoom pictures (Figure 5C2, Supplementary Figures SC16A3). The inverse procedure leads to estimated parameters, one for each ROI, that are compared to the original ones (as defined over each ROI according to Eqs 1, 2) in Table 2 The plasma volume fractions range from 0.08 for zone IV to 0.2 for zone I, overestimated on average for both models by 27%. The rescaled flows range from 5 10^7^ *μm* ^3^/*s* for zone IV to 10^8^ *μm* ^3^/*s* for zone I, with an average error for both models of 13%. In the inference procedure, the plasma-tissue exchange parameter was not set *a priori* to zero, and was recovered low for both models (at least four order of magnitude lower than the flow). Despite these errors, the four zones are ranked in the same order as the original data in terms of plasma volume fraction and flow, for both models: the region with the highest flow and plasma volume fraction is the one that contains the tumor.

**TABLE 2:** Data (direct problem) and estimated parameters for Brix II and DE-Tofts for each ROI I, II, III and IV of Figure 5. *K*_*PS*_ and *F* are in *μm* ^3^/*s*. ROI size: 709 voxels, ≈2mm diameter and $\approx 0.15\;mm^{3}$ volume V. Relative errors ((data-recovered)/data) are reported for *ϕ*_*P*_ and *F*. For *K*_*PS*_, since data is 0, no relative error can be reported.

Zone	Data			Brix II			DE-tofts			Error brix II		Error DE-Tofts	
	*ϕ*_*P*_	*K*_*PS*_	*F*	*ϕ*_*P*_	*K*_*PS*_	*F*	*ϕ*_*P*_	*K*_*PS*_	*F*	*ϕ*_*P*_	*F*	*ϕ*_*P*_	*F*
I	1.67E-01	0	1.26E+08	2.05E-01	2.71E+03	1.08E+08	2.00E-01	9.30E+03	1.12E+08	0.23	0.14	0.20	0.11
II	7.61E-02	0	6.24E+07	9.78E-02	1.90E+02	8.27E+07	9.70E-02	1.20E+03	8.16E+07	0.29	0.33	0.27	0.31
III	6.63E-02	0	5.24E+07	8.50E-02	9.48E+02	5.15E+07	8.35E-02	3.04E+03	5.32E+07	0.28	0.02	0.26	0.02
IV	6.10E-02	0	4.73E+07	8.12E-02	3.80E+02	5.09E+07	7.99E-02	1.96E+03	5.09E+07	0.33	0.08	0.31	0.08

##### 3.2.1.4 The three dimensional case

Finally, a 3 d example with an intravascular agent is presented, where the vascularized tumor tissue is built in the same manner as for the previous 2 d case but with now a 3 d network graph (Figure 5D). In this example, the vascular tumor rim is a thick sphere: 2 d central cuts of the resulting flow and plasma volume fractions show the main vessels, the tumor rim and necrotic core. When the inverse procedure is performed for each voxel (as in Supplementary Appendix SC5), only Brix II gives reasonable results, i.e. the recovered parameter maps show the same structures as the original ones (Figure 5D1, right vs. Supplementary Figures SC16B1 right). Despite the Euclidean distance rescaling of the flow, one can see the influence of the distance to the feeding artery in the quality of the results. For the DE-Tofts model, this influence gets rapidly worse (Supplementary Figures SC161, right). In such conditions, considering ROIs of the size of a typical DCE-MRI voxel (0.3 mm*0.3 mm*3 mm) leads to a major blur of the parameters (Figure 5D2, Supplementary Figures SC16B2). In the original data, the larger vessels and tumor zone cannot be numerically distinguished in the flow, but only the tumor zone in the plasma volume fraction can. The same patterns are seen in the Brix II recovered parameters. As expected by the results at the voxel level, the DE-Tofts model barely recovers the tumor zone in plasma voume fraction map (Supplementary Figures SC16B2, right).

In summary, the simulation results show that plasma volume fraction, exchange rate and the flow can be reasonably well estimated, the latter by scaling the flow rate with the Euclidean distance between feeding point and ROI. This tendency holds true in *d* = 1, two for the Brix II and DE-Tofts models. In 3 d the Brix II model performs better than the DE-Tofts model. Generally, the quality of parameter inference decreases with *d*.

#### 3.2.2 Extravascular agents

Next, extravascular cases are considered. Direct simulations for four different levels of permeabilities *P* are carried out in one and quasi-3d cases, to test the influence of permeability level on the recovery procedure. For the quasi-3D cases, the initial maps are inherently composed of varying vessel density, flow and exchange parameters. Each time the inference procedure is tested for every voxel with the 2 TK models.

##### 3.2.2.1 The one dimensional case

Different from the purely intravascular situation (Figure 5A) now the exchange rate can be non-zero. The default value is taken as *P* ∼ 0.1 m/*s* and in the simulations a range of permeability values is considered (see Section 4.3 on the choice of these values). In the direct simulations, the permeability is varied by several orders of magnitude, resulting in the CA tissue concentration deviating more and more from the AIF, both close and far from the inlet (Supplementary Figures SC17): the first peak gets lost in the signal that continues to rise much later than the AIF, and significantly higher than the first peak contrarily to the AIF. The different models can in general well capture these shapes. The plasma volume fractions and exchange rates (Supplementary Figures SC18 bottom and middle respectively) are well estimated with the different models, but this becomes increasingly more difficult with increasing permeability and distance to the inlet, eventually failing for *P* = 1 *μm*/*s*. The apparent flow parameters estimated by the different models differ from the input flow. Flows can be recovered as in the previous section by rescaling with the length of the path from the feeding artery (Supplementary Figures SC18). But this is getting more inaccurate as permeability increases. In fact, the models become unable to capture the signal shape for slow apparent flows typically recovered in voxels far from feeding artery (Supplementary Figures SC17 third row) for *P* = 1 *μm*/*s*. Because of boundary conditions at the entrance and exit of the tube, an artefact can occur for these voxels (Supplementary Figures SC17).

##### 3.2.2.2 The multi-dimensional case

**FIGURE 6:**
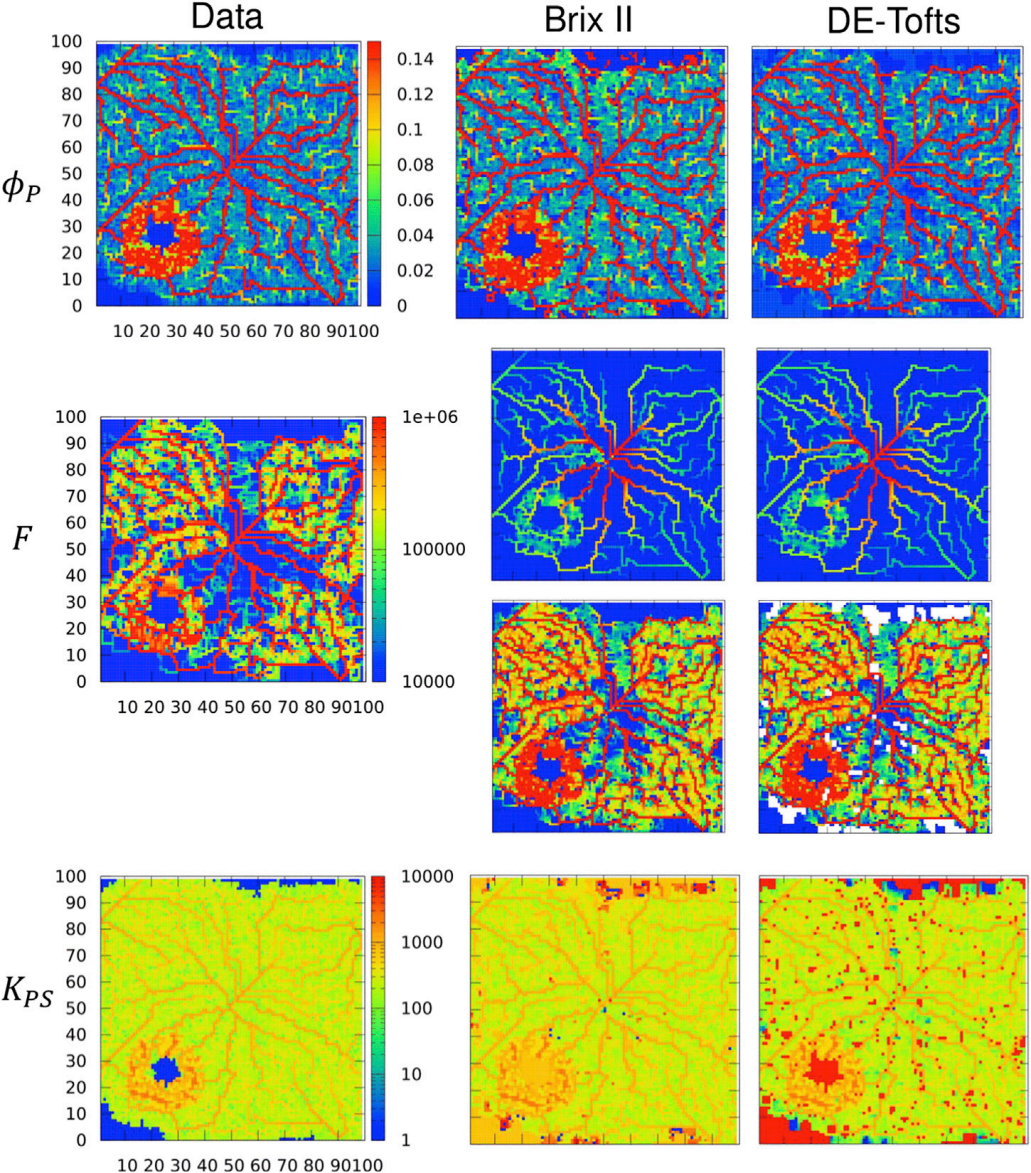
Inferred TK model parameters from contrast-agent time course in quasi-3d. Columns: left = data, center = Brix II, right = DE-Tofts, vessel permeability is *p* = 0.1 *μm*/*s*. First row: vascular density *ϕ*_*P*_, second row: flow, third row: rescaled flow, fourth row: exchange rate *K*_*PS*_. Voxel size: (60 *μm*)^3^, domain: 100 × 100 × 1 voxels. *F* and *K*_*PS*_ in *μm* ^3^/*s*. Same scale bar for all *ϕ*_*P*_, *F*, *K*_*PS*_.

**FIGURE 7:**
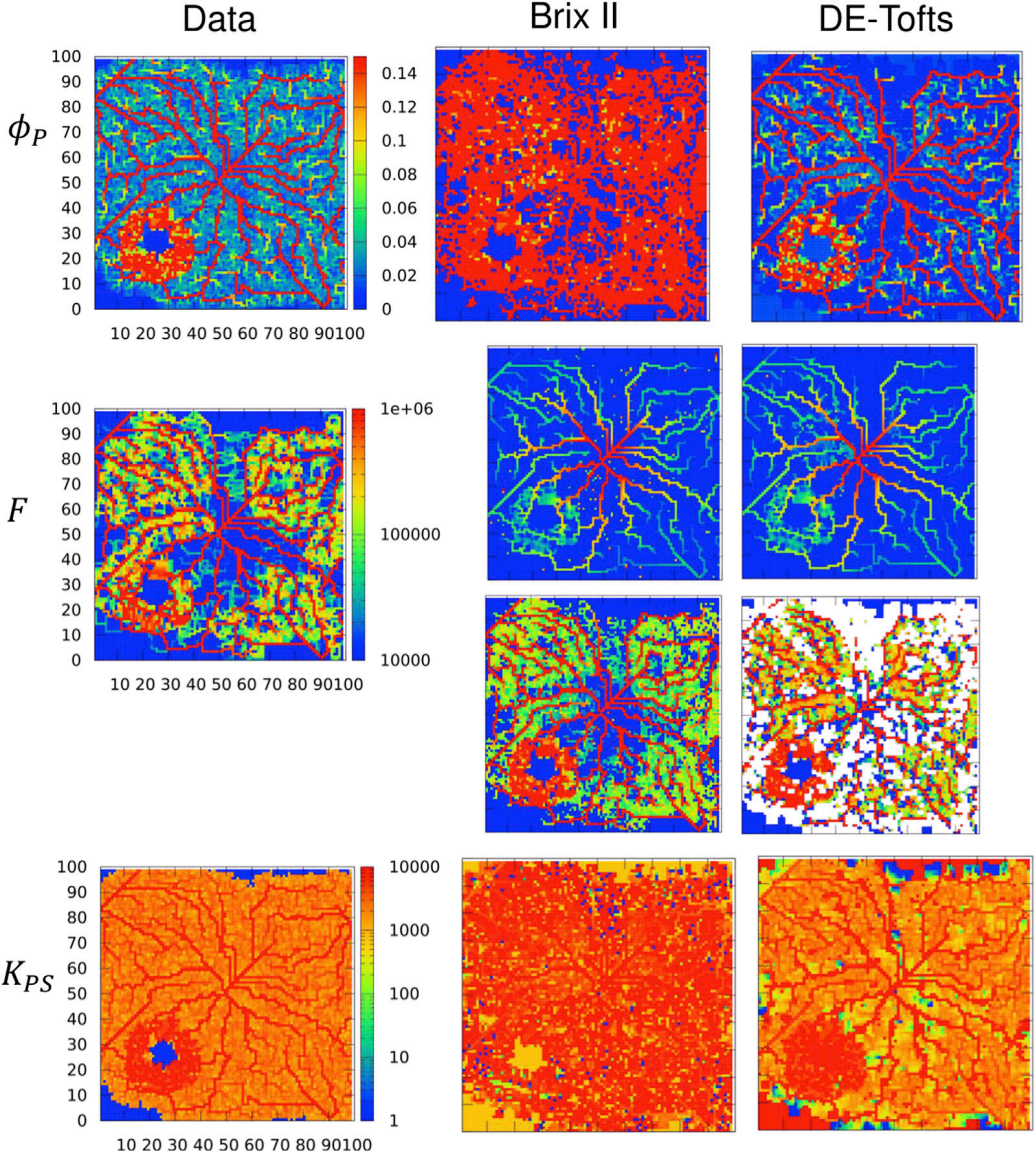
Inferred TK model parameters from contrast-agent time course in quasi-3d. Columns: left = data, center = Brix II, right = DE-Tofts, vessel permeability is *P* = 1 *μm*/*s*. First row: vascular density *ϕ*_*P*_, second row: flow, third row: rescaled flow, fourth row: exchange rate *K*_*PS*_. Voxel size: (60 *μm*)^3^, domain: 100 × 100 × 1 voxels. *F* and *K*_*PS*_ in *μm* ^3^/*s*. Same scale bar for all *ϕ*_*P*_, *F*, *K*_*PS*_.

Next the 2 d case is considered as in Figure 5B, but this time for an extravascular agent. Note that even though the vasculature graph is embedded in 2 d (plane), the simulation is 3 d in the sense that diffusion in the extravascular space occurs everywhere in the domain, like in a 3 d slab of tissue (2 d plane that includes the vasculature over a 6 mm*6 mm square and a small height of 60 *μm*). We refer to this case as ‘quasi-3d’. Different permeabilities are modelled keeping the other parameters unchanged. Thus the flow and plasma volume fraction parameter maps are the same for *p* = 0.1 *μm*/*s* (Figure 6) and *P* = 1 *μm*/*s* (Figure 7) as for the intra-vascular agent described above (Figure 5, Supplementary Figures SC15). The resulting plasma-tissue exchange coefficient by contrast increases with an increasing permeability, making apparent the necrotic zone (very low *K*_*PS*_), the larger blood vessels and the tumor rim (very high *K*_*PS*_). The measured concentration *c*(*t*) in each voxel, solution to Eqs 1, and, is then the input to the inverse procedure. We do not show *p* = 0.01 *μm*/*s*, because the results are as close to *P* = 0 *μm*/*s* as in the 1 d case. For *p* = 0.1 *μm*/*s* (Figure 6 two last columns), the inferred plasma volume fraction is well captured by both models. The rescaled flow and plasma-tissue exchange coefficient are also well recovered, except in zones where there are no blood vessels. For *P* = 1 *μm*/*s* (Figure 7 two last columns), the situation is markedly different: BrixII is the only one to recover the structures of the flow maps but not the other two parameters, whereas with DE-Tofts, the map structures of plasma volume fraction and *K*_*PS*_ (where there are blood vessels) are captured but the flow structures are very degraded.

## 4 Discussion

The main contribution of the paper is the proof of concept presented in Figure 1, which is based on spatial *in silico* perfusion images. We constructed *in silico* vasculatures to solve the direct problem of contrast agent perfusion (intra-vascular transport, intra-extra-vascular exchange and diffusion within interstitial space) for evaluation of parameter estimations from different tracer kinetics models used in dynamic contrast-enhanced (DCE) perfusion imaging. The transport in and out of the 2 d or 3 d vascular networks is modeled by multiphase equations that use as an input a 1 d model of blood flow inside the vessels, the latter limiting the computational cost. Here, as a proof of principle we showed the evaluation procedure of Brix II and DE-Tofts with *in silico* perfusion data of a contrast agent after a bolus injection. For the inverse procedure, six ground truth 1D cases (varying permeability and noise levels), four ground truth 2D/quasi-3D cases (varying permeability levels), and one ground truth 3D case were carried out as direct simulations. For 2D/3D cases, vessel volume fraction, flow and exchange parameters were inherently varying locally. Different inverse procedures were tested each time - i.e. varying ROI location and size, and TK models. For each 2D simulation, the inverse problem was performed for the 10^4^ voxels, and in 3D, the inverse problem was performed for 2.5 10^5^ voxels and for the coarser case for 200 ROIs.

### 4.1 Discussion on the generated architecture and DCE-images

The direct model parameters are not always known (see section 2.2.3), and hence to assess if they are representative of real tissues is a challenge. In healthy vasculature (e.g. Figure 6), *K*_*PS*_ = 200 *μm* ^3^/*s*, and thus *K*_*PS*_/(*ϕ*_p_*V) = 1.4 min^−1^, which is close to the value of 0.2 min^−1^ reported in (Brix et al., 2004). In the tumor region in Figure 6 where parameter inference is successful for *p* = 0.1 *μm*/*s*, *K*_*PS*_/*V* = 0.3 min^−1^, which is close to the values reported by (Brix et al., 2009) and used as baseline in (Zwick et al., 2010) *K*_*PS*_/*V* = 0.2 *ml* min^−1^. *g* ^−1^. Regarding flows, the obtained healthy and tumor flows *F*/*V* ≈ 15–150 min^−1^ on a voxel level and *F*/*V* ≈ 20–40 min^−1^ for the ROI cases (Table 2) are higher than the values reported in (Brix et al., 2004; Brix et al., 2010) but the increase by a factor of 2–10 from healthy to tumor values is reasonable. Moreover, a number of healthy perfusion parameters compared well with literature data over several vessel diameters and tree architectures (Section 3.1.1).

For the tumor vasculature and DCE-images, qualitative comparisons were made in Sections 3.1.2, 3.1.3 to further assess the realism of simulations. Besides, the change of shape of the concentration over time, when increasing permeability (e.g. Supplementary Figures SC17) is similar to that reported in Figure 2B for a lung tumor or also in (Brix et al., 2004; Zwick et al., 2010).

The model can be readily adapted if more precise parameter information is available, concerning each component of the pipeline, including the vascular network generation and their related functional values (if e.g. more detailed experimental studies simultaneously providing information on micro-vascular network architecture and function become available).

### 4.2 Discussion on the parameter inference assessment

Regarding the interpretation of the perfusion parameters, overall parameter inference through TK models successfully detected the tumor area, with increased vascular density, flow and exchange rate (leakage). Studying the influence of space, we thus propose the following parameter inference procedure:

• directly interpret the plasma volume fraction,
• where the former is non-negligible, consider the plasma-tissue exchange-rate recovered value,
• rescale the flow-rate by the distance of the voxel to the contrast-agent arterial input function location.

Similarly to (Zwick et al., 2010), we found that the vessel volume fraction was a very robustly recovered parameter in all cases: with or without noise, in multiple dimensions, varying permeability. For the perfusion parameter (flow) however, we showed how results from DCE-imaging parameter estimation need to be interpreted with care. This is especially true the further the voxel is from the feeding artery: in this case, the flow is more underestimated compared to its true value.

We proposed to rescale the value with the distance to the feeding artery. Actually, the real distance traveled by the contrast-agent should be taken into account, so by rescaling with the true graph distance (if accessible) instead of by the Euclidean distance to the feeding artery the parameter inference would be expected to further improve. A simple estimate of this distance may be obtained by computing the average time of transport through the identified network and the blood flow, yielding a flow-related distance. The *distributed parameter models* based on the 1 d cylindrical capillary-tissue system are attempts to take this spatial component into account but they have not gained practical use (Koh et al. (2011); Sourbron and Buckley (2013)), probably because the overall network leads to more complex spatial influences (see discussion on dispersion below). A TK model that take only diffusion fluxes into account but with a known diffusion parameter, has been shown to improve the inverse procedure compared to the standard Tofts model for a direct model that does not included advection but focuses on diffusion (Pellerin et al., 2007).

For permeability or the plasma-tissue exchange rate *K*_*PS*_, the recovered value was reasonable if the intrinsic permeability *P* is not too high compared to *F*/*S* i.e., if the dimensionless number *P*/(*F*/*S*) = (*PS*)/*F* = *K*_*PS*_/*F* was not too high (see for example Supplementary Figures SC17). The value should be discarded though when the vessel volume fraction or perfusion are close to zero, because in this case the signal intensity is not sensitive to this parameter. For the DE-Tofts model, this is consistent with the interpretation of *K*_*trans*_ as being *K*_*PS*_ only in high flow regime (Tofts et al., 1999).

The 2 d simulations (Figures 6, 7, quasi-3d in that they have been performed in a 2 d slice of thickness 60 *μm*) suggest a possible strategy by combining both BrixII and DE-Tofts models to infer the parameter set composed of plasma volume, plasma-tissue exchange rate and flow (even if unusual for DE-Tofts). If both give similar results for the plasma volume, the permeability is likely to be not too high, and both models may be used to estimate all three parameters. In our simulations, very similar results were obtained for zero up to moderate plasma-tissue exchange rates. If both give different values, this may indicate a high permeability. In that case, BrixII gives reasonable estimates of the flow and plasma-tissue exchange rate, while DE-Tofts better captures the plasma volume fraction. However, a complex 3 d network architecture fed by a distant feeding vessel (as in Figure 5) surprisingly hindered a reliable parameter estimation in particular by the DE-Tofts model even for zero vessel permeability, indicating further need of extensive studies for 3 d vasculatures.

This work also highlights the dispersion effects introduced by heterogeneous vessel networks. Although interesting (Bassingthwaighte and Goresky, 1984), this is another challenge for parameter estimation: the current tracer kinetics models do not contain dispersion per say, and thus the recovered parameters are affected by it. Nevertheless, in 2d, the ranking of the different zones (more or less vascularized and with functional flow) could be distinguished. More work is needed in this direction. In particular, one could use the direct problem to model dispersion in a discretized vascular network in a similar approach as Koch et al. (2020).

In fact, as the size of the ROI increases, dispersion and other effects e.g. due to several feeding vessels entering and leaving the ROI, might affect the overall ROI signal. As a consequence, the recovered ROI parameters will be effective, and may or may not relate to the microscopic parameter as in Eq. 8. The preliminary results on the 3 d case indicate that varying ROI sizes or image resolution impacts the structures that can be identified. To study how ROI parameters scale with ROI size will allow to compare their values with those reported *in vivo* at typical image resolution. In that sense, the proposed framework can be used to study the effects of the perfusion image resolution on understanding the underlying microscopic structure and function.

### 4.3 Current limitations

Several simplifications have been made in the current framework that could be improved as future work. First, the vascularization is built on a regular lattice and thus the vessel diameter is limited by the lattice constant. This constant is 60 *μm*, reflecting a reasonable orthogonal minimal capillary distance of the order of the diffusion length of oxygen in tissue and typical values determined in tissue micro-architectures but the precise value would depend on the particular tissue under consideration (Pries and Secomb, 2011). To consider larger vessels and thus larger trees, generation of the vasculature in lattice-free space could be favorable even if computationally more expensive. Here the choice was made to have tumors embedded into an otherwise normal hierarchical vasculature, and as a consequence these tumors are small. Although tumors of similar sizes (2–4 mm) are found histologically and on DCE-MRI data (Figures 2C,D,G), studying larger tumors with the same proof of concept would be a valuable future complement to the current work.

Furthermore, the extravascular compartment does not distinguish between intracellular and extracellular spaces, to reduce the number of estimated parameters. As long as the cells do not take up the CA as well, this is not changing the qualitative kinetics meaning only the precise concentration values may change. I.e. it does not affect the conclusions of this work. In all cases the same workflow described in this work can be pursued to study the precise parameter values of the different inference models.

Finally, additional physical effects could be implemented in the direct problem such as tumor filtration as done by Penta et al. (2015), without changing the workflow.

### 4.4 Strengths of approach

The model is flexible since one can vary architecture and function of the vascularization (control of features such as regional microvascular density, necrotic core, etc.) and see their effects in synthetic images. This framework allows to test hypotheses supported by that another recent work points into similar directions (Nuha Abdul et al., 2021). Different other TK models can also be tested within the same framework. Moreover, such model permits to study the ambiguity caused by different tumor vasculatures (highly permeable *versus* high microvessel density) leading to similar perfusion images (bright tumor area, dark necrotic core) and their characteristic differences (temporal occurrence as e.g. delayed clearance in case of high permeability). In that sense, it brings function to histological data. While histological slices (see an example in Figure 2G) provide direct tissue information, they only give vessel density information or, after challenging image analysis, architecture, but not functional information. Moreover, often part of the vessels found in pathological specimen are not perfused. In-silico modeling may thus provide an interesting complement to interpret such data.

Different injection procedures (bolus injection *versus* gradual administration) could be easily studied along with other implications for diagnosis (temporal resolution and duration of acquisition).

## 5 Conclusion

To conclude, this work presents the construction of a pipeline to investigate the link between DCE images and the underlying tissue microstructure and function, and demonstrates its possibilities on a variety of cases. This pipeline is based on a spatial model of vascularization and contrast-agent transport in and out of the vasculature. The direct model parameters for healthy and tumor zones are based on literature values and varied for sensitivity analysis of the *in silico* images to model inputs. Overall the parameter inference from the TK models typically applied to characterize DCE images, successfully detects the tumor features compared to the surrounding tissue. The results of the parameter inference indicate that the inference for both Brix II and delay-adjusted extended Tofts models is robust for low permeability and little dispersion due to network heterogeneity. The plasma volume fraction and the vascular-tissue exchange rate where the former one is non-negligible, are directly accessible while the recovered flow parameter needs some reinterpretation. Rescaling the flow by the ROI distance to the feeding artery significantly improves its estimation, the Euclidean distance being a work-around up to a certain point to estimate the time the injected CA volume needs to travel to the ROI. The results indicate the importance of taking explicitly into account space in inverse models. This study lays the foundation for better studying the effect of coarse-graining (or ROI sizes) in such dynamic imaging modalities and also for better interpreting histological data since they only provide structural but not functional parameters.

## Data Availability

The original contributions in the study are publicly available. This data can be found here: https://gitlab.inria.fr/simbiotx/dyna-imaging-mod.

## Nomenclature

***V***: Graph vertex set
***E***: Graph edge set
***G* = (*V*, *E*)**: Graph
***l*_*i*,*j*_**: Length of edge (*i*, *j*)
***r*_*i*,*j*_**: Radius of edge (*i*, *j*)
***η*(*r*_*i*,*j*_)**: Apparent viscosity of edge (*i*, *j*)
$G_{i,j}=\frac{\pi }{8}\cdot \frac{r_{i,j}^{4}}{\eta \left(r_{i,j}\right)\cdot l_{i,j}}$: Hydraulic conductance of edge (*i*, *j*)
**Δ*p*_*i*,*j*_ = *p*_*i*_ − *p*_*j*_**: Pressure difference on edge (*i*, *j*)
***f*_*i*,*j*_ = *G*_*i*,*j*_ ⋅Δ*p*_*i*,*j*_**: Flow rate on edge (*i*, *j*)
$\tau_{i,j}=\frac{4\eta \left(r_{i,j}\right)f_{i,j}}{\pi r_{i,j}^{3}}$: Shear stress on edge (*i*, *j*)
$\phi_{P,i}=\frac{V_{P,i}}{V_{i}}$: Plasma volume fraction in voxel of volume *V*_*i*_
***ϕ*_*I*,*i*_ = 1 − *ϕ*_*P*,*i*_**: Extra-vascular volume fraction in voxel of volume *V*_*i*_
***F***: Flow rate in voxel of volume *V*_*i*_ (volume flow rate)
***c* ^*m*^**: Microscopic concentration
***D* ^*m*^**: Diffusion coefficient
***H* ^*m*^**: Membrane thickness
***S* ^*m*^**: Membrane surface
****J** = −*D* ^*m*^∇*c* ^*m*^**: Diffusion flux
**v** ^**m**^: Microscopic velocity field of the fluid containing the contrast agent
***c*_*P*_**: Plasma macroscopic concentration
***k*_*PS*_**: Volumetric macroscopic membrane exchange rate
$k_{PS}^{i}$: Volumetric macroscopic membrane exchange rate in a voxel *i*
$K_{PS}^{i}$: Exchange rate in a voxel *i*
$P_{i,j}=\frac{D_{i,j}^{m}}{H_{i,j}^{m}}$: Permeability of edge (*i*, *j*)
***P***: Permeability constant over a region for all its vascular edges
**v**: Macroscopic velocity field
***c*_*A*_, *C*_*A*_**: Arterial input function concentration
***c*_*I*_**: Extra-vascular macroscopic concentration
***D*_*I*_**: Diffusion coefficient in extravascular domain
***c*_*i*_ = *ϕ*_*Pi*_ *c*_*Pi*_ + *ϕ*_*Ii*_ *c*_*Ii*_**: Total concentration in voxel *i*
***C*_*P*_**: Brix II/Extended Tofts plasma concentration
***C*_*I*_**: Brix II/Extended Tofts extravascular concentration
***K*_*trans*_**: Transfer constant in extended Tofts model
***t*_0_**: Tofts delay
***β***: Set of parameters to fit
***S*(*β*)**: Quadratic fitness function to minimize
***F*_*recov*_**: Estimated flow rate from compartment models
