# Supplementary material for: A proof-of-concept pipeline to guide evaluation of tumor tissue perfusion by dynamic contrast-agent imaging: Direct simulation and inverse tracer-kinetic procedures
Source: Front Bioinform. 2023 Apr 13;3:977228. doi: 10.3389/fbinf.2023.977228 (PMC10135870; doi:10.3389/fbinf.2023.977228)
Supplement: Supplementary file 5 [file Presentation1.pdf]

Appendix A. More precise information on background on perfusion imaging and its modeling steps

Appendix A.1. Perfusion imaging appendix

In dynamic contrast enhanced (DCE) perfusion imaging, the image intensity at each voxel, varying in time is at best interpreted based on models that were constructed to represent as simply as possible the underlying structure and function of the imaged tissue. DCE perfusion imaging (DCE-MRI, DCE-CT, DCE-SPECT, DCE-PET or DCE-ultrasound) has shown great potential to non-invasively assess cancer development and its treatment, even short term (Palmowski et al., 2008b; O'Connor et al., 2011; Lassau et al., 2011).

In these imaging modalities, a contrast agent is injected in the blood stream, which when it spreads in the vasculature, and for extravascular agents also permeates outside of it in-between tissue cells, gives rise to a signal representative of its overall concentration in the tissue (see Fig. 2a,d for an example of DCE-MR image and b,f for an example of dynamic signal in a region of interest). Even at small sizes, tumors can be detected by such imaging modality (Fig.A.8).

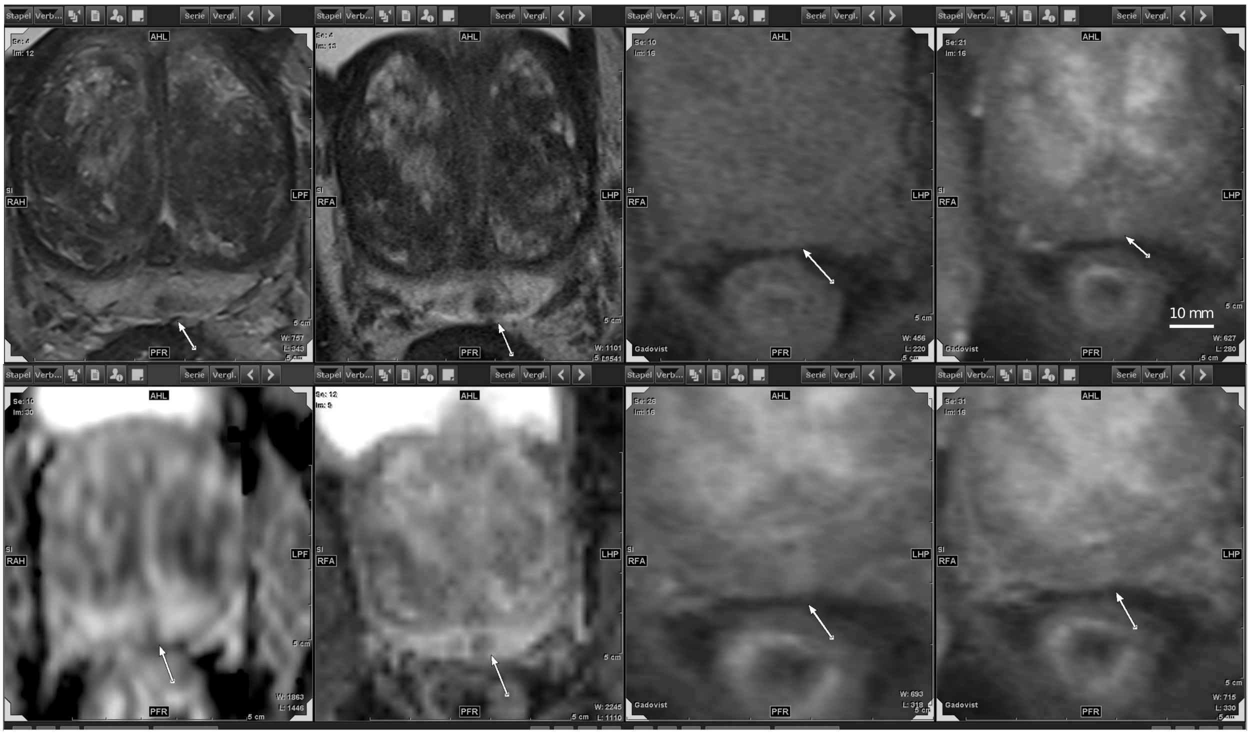

Figure A.8: **Multimodality imaging of a small prostate tumor** Tumor of 3.8mm already detectable by non-invasive imaging (arrows). a: T2-MRI image.b: DWI-MRI ADC map. c: sequence of DCE perfusion imaging (10, 21, 26 and 31 s) with time resolution of 4.5 s.

Appendix A.2. Modeling background appendix

As explained in the Introduction, a pipeline is proposed (Fig.1): in-silico vasculatures are here constructed to solve the direct problem of contrast agent perfusion with spatio-temporal coupled multiphase partial differential equations, which model intra-vascular transport, intra-extravascular exchange and diffusion within the interstitial space. The inverse problem of estimating morphological and functional parameters (for the present work, see section 2.3.2) from different pharmacokinetic models (ordinary differential equations) can then be evaluated from these in-silico contrast-agent perfusion data. For each of these building blocks, a number of aspects have already been addressed in published references, that are here recalled in more depth than in the introduction.

The first step in this process is the modelling of blood flow in realistic blood vessel networks in the context of tumor. At the time-scale of image data acquisition, the blood vessel network can be considered as static, while it

should retain the hallmarks of cancer vascularization (Bergers and Benjamin, 2003; Vaupel, 2004; Hanahan and Folkman, 1996), namely angiogenesis and necrosis. The corresponding modelling work has focused on detailed interplay between angiogenesis and tumor growth (mainly in 2d, but in a few works in 3d as well) (Lee et al., 2006; Macklin et al., 2009; Drasdo et al., 2010; Perfahl et al., 2011), with non-hierarchical networks that represent flow within the tumor area. The question of hierarchical network formation has been addressed in 2d (Secomb et al., 2013). Such interplay has been modelled with arterial trees of 3 Strahler generations in the context of brain tumor (Cai et al., 2016). To get the tumorous network from image analysis and compute blood flow in it presents a number of challenges such as difficult identification of arterial and venous trees, and is thus rare (Stamatelos et al., 2014). As noted in Rieger and Welter (2015), representing hierarchical arteriovenous networks is however important to study tumor vascularisation, and the present work is largely inspired by Welter et al. (2008); Welter and Rieger (2010).

Based on blood flow, the transport of contrast agent or drugs inside these vessels and outside can then be computed. The spread of such compounds within vessel networks has been modelled mainly intravascularly. In particular, McDougall et al. (2006); Welter and Rieger (2010) studied the tumor drug access (i.e. the amount of drug going through the vascular network) in an angiogenesis model, in 2d and 3d respectively. This later flow and drug transport based on an arteriovenous network has been extended in a comprehensive model that includes both for the fluid and the drug the multiphasic interstitial space, including lymphatic removal but drug transport is not accounted for in the vessel network (Welter and Rieger, 2013). In a similar model but without the arteriovenous network, in 2d (Wu et al., 2014) and in a 3d slab (Cattaneo and Zunino, 2014), different therapeutic injection schemes were evaluated. Penta et al. (2015) derived a homogenized theoretical model taking into account blood flow, interstitial flow and drug transport. The tumor was modeled through the physiological fluxes crossing the vessel's membrane (e.g. by choosing a higher hydraulic conductivity of the vessel wall). Mescam et al. (2010) studied in liver tissue a multiscale model of blood flow and contrast-agent spread, to better understand DCE-MRI images in the presence of tumor. The macro-model considers the larger arterial and venous trees, connected to micro-scale units where capillaries are not explicitly represented, but the contrast-agent spread is finely represented by a 1d 5-compartment model for each micro-unit (close to the capillary-tissue exchange unit model of (Bassingthwaighte et al., 1989), see below), which gives rise to a signal at the macro-level by integration over the capillary length. The focus there is to generate and understand DCE-MRI images at the whole-organ level. For a more in-depth modelling review of tumor vascular remodelling and drug transport, we refer to Rieger and Welter (2015) and references herein.

Once a dynamic signal over time is generated from the spread of the contrast agent, the next issue is to infer some parameters from it that reflect the underlying tissue. The signal is interpreted at the image voxel level or more commonly in a whole region of interest (ROI), typically a region that is thought to be the tumor or a non-tumor zone, or dividing the tumor with an *onion-peeling* approach (Yang and Knopp, 2011). As explained above, in the dynamic imaging community, this step is done with a variety of tracer kinetics models. Most of them do not take fluxes (such as transport of matter in space) explicitly into account, except for example, *distributed parameter models* such as the Johnson and Wilson model (or tissue-homogeneity model), a 1d capillary exchange model that is not used in practice as it cannot be solved easily (Sourbron and Buckley, 2013). Many models fit into the general theoretical framework proposed by (Sourbron, 2014) (partially building upon ref. (Pellerin et al., 2007)) that permits to create tracer kinetic schemes from a set of simple graphical modular process elements and map them to a mathematical description in terms of equations. The processes covered are (pseudo-)diffusion, advection, leakage, absorption, decay and sources of tracer, hence belonging to the class of multiphase diffusion-advection-chemical reaction equations. Such equations are usually good approximations if the tracer concentration is very low i.e., the tracer molecules are highly diluted. In fact, the signal can easily be analysed by extraction of semi-quantitative parameters (time to peak, the area under the curve, maximum slope, etc.), without being specific for e.g. blood flow, but rather indirectly related to the underlying tissue structure. Deconvolution methods on the other hand relate the signal to a residual function: its area under the curve and peak are associated with blood volume and blood flow per volume of tissue respectively, and their ratio gives the mean transit time (Gauthier et al., 2012; Sourbron and Buckley, 2013). They are especially relevant for intravascular agents, such as in DCE-US (Gauthier et al., 2012) or when the blood-brain barrier is intact and studied with DCE- or DSC-MRI (Sourbron and Buckley, 2013). When the agent can extravasate (such as in DCE-CT and DCE-MRI), since it is usually not locally eliminated except for liver studies which present a particular modelling challenge (Forsgren et al., 2014), models usually consist of mass balance and exchange of the contrast-agent in two

compartments, one for blood and one for the extravascular space (Lee et al., 2003). Often, further assumptions are made such as small blood volume (Tofts model) or high blood flow (Extended Tofts model) (Sourbron and Buckley, 2011). In any case, it is assumed that the feeding concentration to the blood compartment is the same for all voxels, possibly with a delay-parameter (Lee et al., 2003; Sourbron and Buckley, 2013; Koh et al., 2011). This feeding concentration is taken as the arterial or vascular input function, either assumed to represent an average patient one or is measured in the patient on a large artery. For reviews of the subject that include model choices and clinical applications in DCE-MRI and DCE-CT, see (Tofts et al., 1999; Brix et al., 2004; Yang and Knopp, 2011; Koh et al., 2011; Sourbron and Buckley, 2012, 2013; Ingrisch and Sourbron, 2013; Brix et al., 2017).

When the signal comes from in-vivo data, it is difficult to assess if, given all these hypotheses, the retrieved parameters are representative of the underlying tissue. Architectural parameters can be compared to invasive histological data (Zwick et al., 2009; Yin et al., 2018). In-vitro experiments have shed light on this process for DCE-US (Gauthier et al., 2012), DCE-CT (Driscoll et al., 2011) and perfusion imaging with SPECT and PET (Gabrani-Juma et al., 2016). However to construct a representative vascularised tumor phantom at scale is challenging. Comparison to other blood flow imaging measurements modalities have validated its use, e.g. in DCE-CT (Lee et al., 2003), but at the macro-scale. Another way is thus to do it in-silico. Comparison of different two-compartment models of different complexities has been done in (Luypaert et al., 2011; Pellerin et al., 2007). In Bassingthwaighte and Goresky (1984), the signal was generated with a 1d capillary-tissue exchange model and a variety of variations to discuss the impact on signal analysis by deconvolution. The *multiple-path multiple-indicator dilution, 4 region model* (MMID4) (Bassingthwaighte) stems from this work: it takes into account the fact that the contrast agent runs through different capillary-tissue exchange units in parallel as well as arterial and venous trees before and after them, thus allowing generation of up to 20-paths with their own parameters, such as delay and dispersion for transport in the trees. In Zwick et al. (2010); Brix et al. (2010), the MMID4 model was used to generate the signal, varying the input parameters to generate a number of signal curves: the steepest slope method was thus assessed (Brix et al., 2010), as well as how parameters of two simplified models correlate to the tissue input parameters (Zwick et al., 2010). However in both cases, these parameters are only indirectly related to the input parameters.

Appendix B. Material and Methods appendix

Appendix B.1. Vascular network generation

Appendix B.1.1. In-silico vascularization

Vessel graph and voxels. Vessels are represented by a graph $G = (V, E)$ of vessel nodes V connected by vessel segments (cylindrical tubes) $E \subset V \times V$, here on a square lattice (see Fig.B.9) with a lattice constant l. One node can be connected to another one if they are at the adjacent lattice site in x or y or z direction (von Neumann neighborhood). It has at most $2d$ neighboring nodes, d being the lattice dimension. A node is either a root (boundary to the rest of the circulation, root of arterial or venous tree), interior or tip (tree leaves creating capillary connections with other tips). As described later all vessel networks grown from single vessel root nodes are by construction trees, but the vessel network as a whole will form a graph by inter-tip connections. The dual lattice of the vessel lattice (Fig.B.9) describes the spatial partition into voxels, representing the tissue compartments.

Vessel properties. On a vessel graph, vessel geometrical and functional properties are computed as follows. As venous and arterial trees interpenetrate at the capillary level, all neighboring tip nodes form inter-tip connections of capillary size: $r_{i,j} = 4\,\mu m$, if i or j is a tip node. Then the radii of the remaining vessel segments are recursively calculated by

$$r_{i,j}^{\alpha} = \sum_{k,k \neq j} r_{i,k}^{\alpha}. \tag{B.1}$$

since all $r_{i,k}$, $k \neq j$, are already known by construction. The values of α vary between 2 and 3 depending on the vessel size and tissue. A representative value is $\alpha = 2.7$ (Kurz and Sandau, 1997). Different default values for arteries, $\alpha^{art} = 3$, and veins, $\alpha^{ven} = 2.7$ are here chosen, to better fit the asymmetric network properties (see Results and Fig. 3).

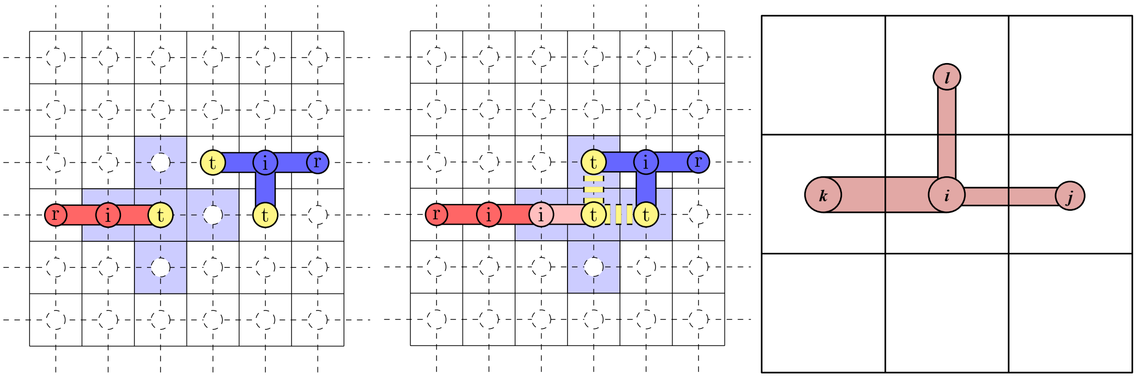

Figure B.9: **Vessel representation.** Left: Vessel nodes (circles) occupy vertices of a square lattice (dashed lines), either as a root (r), interior (i) or tip (t) node. Vessel segments connect a node to neighboring (von Neumann) nodes (pale blue squares for the middle tip node). We distinguish here between vessel (non yellow) or inter-tip (yellow) connections, and among vessels between arterial (red and pink) and venous trees. The dual lattice represents the discretization of the tissue outside of the vessels (solid squares). Left to middle: A tip node is sprouting rightwards inside its von Neumann neighborhood and is forming a new vessel segment (pink). The original tip node becomes an interior node. Right: After the new (k,i) vessel forms, inter-tip connections (i,j) and (i,l) form automatically between neighboring tip nodes.

Blood in each vessel is modeled as an incompressible fluid, with a dynamic apparent blood viscosity $\eta(r)$ that depends on the vessel radius and hematocrit measured in vivo by (Pries et al., 1994). Here we assume a normal hematocrit of 0.45 (Purves et al., 2003) but more sophisticated rheological model in networks could be taken into account (Boissier et al., 2021). In the microcirculation, the Reynolds and Womersley numbers are small, and thus flow is essentially steady, unidirectional, axisymmetric and developed: Hagen-Poiseuille flow is a good approximation if detailed velocity and pressure around each red blood cell are not needed (Fung, 2013). The relationship between flow $f_{i,j}$ through a vessel segment (i, j) of length $l_{i,j} = l$, and pressure of its vessel nodes, $\Delta p_{i,j} = p_i - p_j$, is thus:

$$f_{i,j} = \frac{\pi}{8} \cdot \frac{r_{i,j}^4}{\eta(r_{i,j}) \cdot l_{i,j}} \cdot \Delta p_{i,j} = G_{i,j} \cdot \Delta p_{i,j} \tag{B.2}$$

$G_{i,j}$ is the inverse of the resistance to flow. Applying Kirchoff-law (mass conservation) then relates pressure in node i to pressure in its neighbor nodes j. Pressures can be calculated by solving the linear system:

$$\sum_j G_{i,j} \cdot p_i - \sum_j G_{i,j} \cdot p_j = 0 \tag{B.3}$$

Pressure in the root nodes is given in Table B.3. Flows in the vessel segments are obtained by equation B.2, and shear stresses by:

$$\tau_{i,j} = \frac{\Delta p_{i,j}}{2 \cdot l_{i,j}} \cdot r_{i,j} = \frac{4\eta(r_{i,j}) f_{i,j}}{\pi r_{i,j}^3} \tag{B.4}$$

Parameter	Unit	Value	Description
r^{tip}	μm	4	radius of vessel segment at tips
α^{art}	–	3	power law exponent for arterial vessel segments
α^{ven}	–	2.7	power law exponent for venous vessel segments
l	μm	60	length of vessel segment (lattice constant)
p^{art}	kPa	12	pressure at arterial roots
p^{ven}	kPa	2	pressure at venous roots

Table B.3: Vessel properties for arterial and venous trees.

Appendix B.1.2. Initial vascular networks

In order to construct a vascular network we first place a number of vessel root nodes into some randomly chosen voxels at the domain border. Then, in a naive approach vessels grow and branch randomly until all lattice points are occupied. Here we iterate over all vessel nodes. If at least one neighboring voxel is still not occupied by a vessel node then the vessel node sprouts with probability p^{spr} (Table 1) to one of the free neighbor voxels. The vessel sprouting is done by adding another vessel node to the center of the targeted voxel and connecting it to the originating node of sprouting via a vessel segment. The new node becomes a tip. If the originating node was a tip as well it becomes interior. This process is repeated until all voxels contain a vessel node.

Algorithm 1: Random Vessel Network Creation.

1. For all vessel nodes $v_i \in V$ do
   (a) if v_i has free neighbor voxels sprout with probability $p^{spr} = 0.5$:
      i. add vessel node to randomly chosen free neighbor voxel:
         $V := V \cup \{v_j\}$
      ii. add vessel segment connecting both nodes:
         $E := E \cup \{(v_i, v_j)\}$
      iii. update v_j as tip and v_i as interior
2. Go back to (1.).

Appendix B.1.3. Creation of functional vascular networks

The aim is next to create a blood vessel network consisting of highly functional arterial and venous trees. For this purpose Goedde and Kurz (2001) proposed a Monte-Carlo method which iteratively leads to non-functional vessel collapse (uniquely the tree leaves or tips) and sprouting of functional vessels. They found that rather than flow, it is capillary shear stress which regulates vessel remodeling. The algorithm can be summarized as the following:

Algorithm 2: Shear Stress Homogenization.

1. Calculate the vessel radii (equation B.1), pressure (equation B.3), flow (equation B.2) and shear stress (equation B.4) in vessel network.
2. Compute minimum and maximum shear stresses among all tip segments:
   τ_{min} and τ_{max}.
3. Perform a *vessel collapse/degeneration* for tips randomly chosen with probability
   $p^{deg}_{i,j} = \frac{\tau_{max}-\tau_{i,j}}{\tau_{max}-\tau_{min}}$.
4. Perform 10 iterations of *vessel sprouting* of randomly chosen nodes (see Algorithm 1) with sprouting probability p^{spr} (Table 1).
5. Go back to (1.).

Appendix B.1.4. Tumor vascularization rules

As the higher MVD is mimicked by connecting neighboring capillary ends with n parallel vessels such that each inter-tip connection in Fig.B.9 implicitly represents n vessels, the vessel radii power law (equation B.1) at tips changes to

$$r^{\alpha}_{i,j} = n \sum_{k,k\neq j} r^{\alpha}_{i,k}, \tag{B.5}$$

and the inter-tip Hagen-Poiseuille-law (equation B.2) for pipe flow becomes

$$f_{i,j} = \frac{\pi}{8} \cdot \frac{n r^4_{i,j}}{\eta(r_{i,j}) \cdot l_{i,j}} \cdot \Delta p_{i,j} \tag{B.6}$$

Appendix B.2. In-silico DCE perfusion images

Appendix B.2.1. Derivation of macroscopic multi-phase transport equations

(I) The convection equation in plasma and interstitial space. The contrast-agent (CA) is here considered as a diluted species that does not affect the velocity of the fluid that contains it. At each microscopic point **x** in the blood (Ω_p) or the interstitial space (Ω_I) Ω_m ($m = p, I$), the following CA concentration (c^m) equation can be derived from mass conservation (Pinder and Gray, 2008):

$$\frac{\partial c^m}{\partial t} + \nabla \cdot (c^m \mathbf{v^m}) + \nabla \cdot \mathbf{J} = 0 \ \in \Omega_m \tag{B.7}$$

assuming that no reaction occurs. $\mathbf{v^m}$ is the velocity of the fluid containing CA, and **J** is the diffusive flux of CA relative to the velocity of the fluid.

Here, the diffusion flux is $\mathbf{J} = -D^m \nabla c^m$ according to Fick's first law of diffusion, where D^m is the diffusion coefficient. I.e., the upper equations (B.7) represent two transport (considering convection and diffusion) equations separately for the blood and the interstitial space (domain) whereby the equations do not describe any interaction between the two spaces.

(II) The equation at the interface between plasma and interstitial spaces. Assuming the simplest kinetic formulation across a membrane, here the endothelial wall of the vessels, (Pinder and Gray, 2008), the change in concentration on each side of the membrane, c_1 and c_2, can be described by

$$V_1 \frac{dc_1}{dt} = -\frac{D^m S^m}{H^m}(c_1 - c_2) \tag{B.8}$$

$$V_2 \frac{dc_2}{dt} = \frac{D^m S^m}{H^m}(c_1 - c_2) \tag{B.9}$$

where V_1 and V_2 are the corresponding local compartment volumes, and D^m, S^m, H^m are the diffusion coefficient, surface and thickness of the membrane respectively.

Accordingly, at the interface between the blood vessels and the interstitial space, the blood vessel walls are modelled as a permeable membrane and the diffusion flux across it (normal to the wall) reads as (Fick, 1855b,a; Fung, 1990; Leedale et al., 2020)

$$J = -D^m \nabla c^m \cdot \mathbf{n} = \frac{D^m}{H^m}(c^m - c_I^m) \tag{B.10}$$

where c_I^m the concentration in the interstitial space.

(III) Multi-phase equations: integration of transport in blood and interstitial space (I) and flux between them (II). Multiphase equations can be derived at the macroscopic level by integration of the microscopic equations inside the two 'phases' or tissue components: blood and interstitial spaces. In such model, each piece of tissue in Ω contains both blood vessels and interstitial space of respective volume fractions ϕ_P and ϕ_I. Because the tracer community usually rather refers to plasma, this label will be used instead of blood. Assuming that blood vessel walls are permeable membranes following kinetics of equation B.9 but that no other diffusion or dispersion of the CA occurs inside blood (i.e., the diffusion is negligible compared to the advection as can be quantified by the Peclet number). Mass concentration in plasma and interstitial space finally give raise to equations 1 and 2 respectively.

Appendix B.2.2. Arterial input function

The arterial input function inferred by Parker et al. (2006) from measurements across a population of 113 patients, has the form:

$$c_A(t) = \sum_{n=1}^{2} \frac{A_n}{\sigma_n \sqrt{2\pi}} \cdot e^{-(t-T_n)^2/2\sigma_n^2} + \alpha e^{-\beta t}(1 + e^{-s(t-\tau)})^{-1}. \tag{B.11}$$

The parameters chosen in this study are given in the table B.4.

Parameter	Value	Unit
A_1	0.809	$mM \cdot min$
A_2	0.330	$mM \cdot min$
T_1	0.171	min
T_2	0.365	min
σ_1	0.056	min
σ_2	0.132	min
α	2.100	mM
β	0.169	min^{-1}
s	38.078	min^{-1}
τ	0.483	min

Table B.4: Arterial input function parameters for the studies of this work.

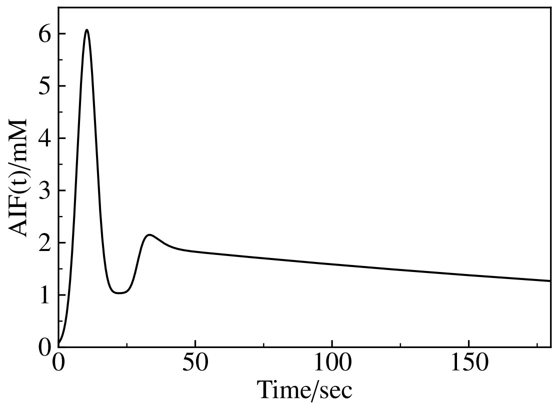

Figure B.10: Shape of the Arterial Input Function (AIF)

Appendix B.2.3. In-silico contrast-agent modeling numerics

The equations (1) and (2) were discretised in space with a classical finite volume scheme: the advection term was treated with an upwind scheme and the diffusion term with a 2nd order mid-point discretization. Solving the resulting ODEs explicitly in time (Forward Euler scheme) leads to the following update during time interval Δt between time step n and $n+1$, for each discrete volume i:

$$c_{P,i}^{n+1} = c_{P,i}^{n} + \frac{\Delta t}{V_{P,i}} \left(\sum_j f_{i,j}^{in} c_{P,j}^{n} - \sum_j f_{i,j}^{out} c_{P,i}^{n} - K_{PS,i}(c_{P,i}^{n} - c_{I,i}^{n}), \right) \tag{B.12}$$

$$c_{I,i}^{n+1} = c_{I,i}^{n} + \frac{\Delta t}{V_{I,i}} \left(\frac{D}{(\Delta x)^2} \sum_j \frac{V_{I,i} + V_{I,j}}{2} \left(c_{I,j}^{n} - c_{I,i}^{n} \right) + K_{PS,i}(c_{P,i}^{n} - c_{I,i}^{n}) \right). \tag{B.13}$$

The time step was chosen such that this explicit scheme was stable for the whole network (Courant et al., 1928). Initial conditions were taken as zero.

Appendix C. Results appendix

Appendix C.1. Construction of a functional vascularization

Fig. C.11 (top) shows a 2d example vascularization resulting from the initial construction step. One can especially notice that the arterial and venous trees stay well separated from each other. Large parts of the vascular networks have low to almost no flow, and, consequently, are not functional at all. On the same figure, one can observe how this randomly initialized vascularization changes during shear stress homogenization. Due to non-functional vessel collapse and the survival of capillaries with sufficiently high shear stress (usually at the interfaces of arterial and venous trees) in the model, the trees of different types (artery/vein) penetrate each other, increasing their common interfaces. As a consequence the inter-tree-connections increase and with them the overall flow. Wall shear stress is then much more homogeneous. This process happens both in the 2d and 3d model.

Appendix C.2. Influence of α exponents in functional vascular tree properties

We elaborate here on a more in depth explanation of the influence of α exponents in the formation of a functional vascular tree.

On one hand, the vessel network is constructed from the capillary tips such that starting with the same tip-vessel diameter independently of α, as done here, the increase of radii from the tips to the roots is the smaller, the larger is α (equation B.1). As a consequence, for a given network topology, all vessel diameters including the root diameter are the smaller, the larger is α, hence the flow resistance that behaves as an inverse power of the vessel diameter (equation

B.2) increases with increasing α. This 1rst effect is counter-balanced by the fact that an exponent of $\alpha = 3$ leads to a maximally homogenized wall shear stress, while $\alpha < 3$ result in more frequent vessel collapse (2nd effect). This is a consequence of Murray's law stating that the observed radii are such that the work for transport and maintenance is minimized, leading to an exponent of 3 for laminar flow, which in turn homogenizes wall shear stress. As in-line with this argumentation, the shear stress-homogenization algorithm (algorithm 2 in Appendix B.1.3) collapses more vessels with increasing heterogeneity in the shear stress (i.e., the smaller is α), the branching tree appears sparser with decreasing α thus increasing the flow resistance with decreasing α. The simulation result indicates that the 2nd effect dominates the first effect.

For the same reason, when increasing the exponent, flow decays more steeply with diameter (from root to tips, Fig.3, 2nd row). For wall shear stress (equation (B.4)) and velocity (Fig.3, 1rst and 3rd rows, respectively), given that they are both ratios of flow and diameter to a positive power, there is competition between increasing flow (from the 2nd effect) and increasing diameter (from the 1st effect). This explains the variability of decay with diameter responses. Thus the functional tree properties depend on the choice of α.

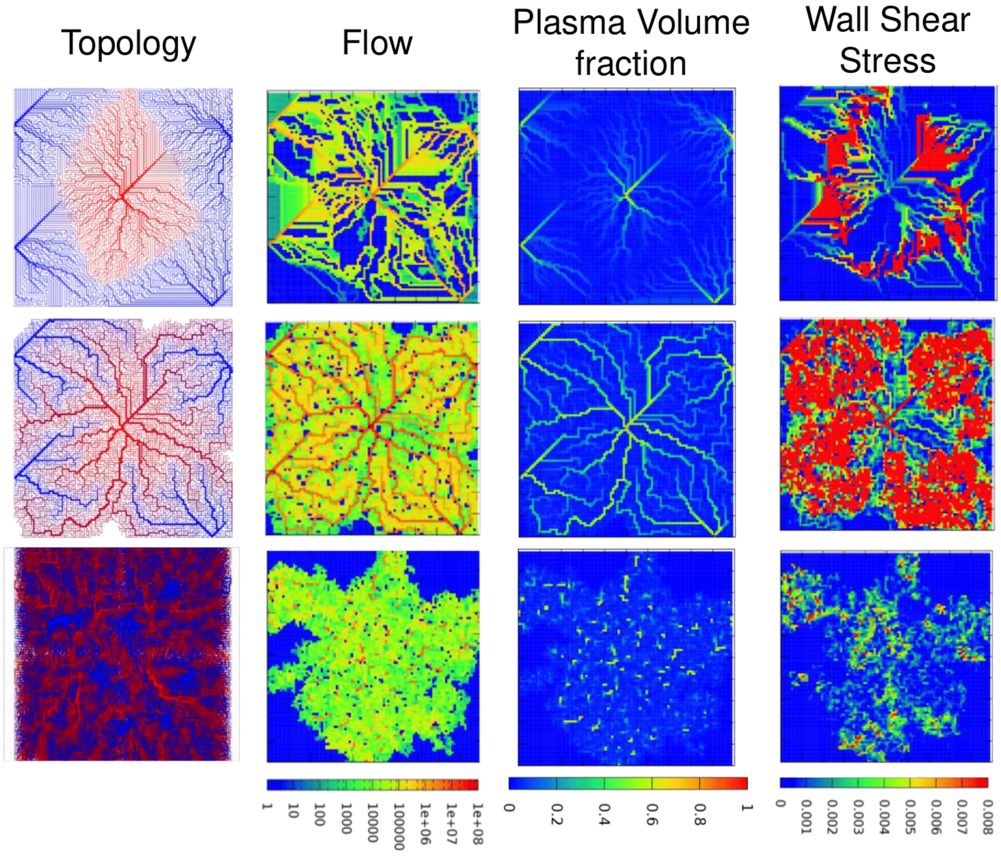

Figure C.11: **Shear stress homogenization for the reference tissue (no tumor).** Top: vascular networks are first grown by randomly sprouting vessels on a 100x100 lattice-cite domain. From left to right: network topology colored by pressure (blue=low pressure at 2kPa, red=high pressure at 12kPa), and parameter maps for blood flow F ($\mu m^3/s$), vascular volume fraction ϕ_P and wall shear stress τ ($g/(\mu m s^2)$). Middle: maps after 100 iterations of shear stress homogenization where the interfaces between venous (red) and arterial (blue) vessel trees increase. Bottom: cut of 3d model (100x100x100 lattice-cite domain) of the same maps. Lattice constant: 60μm.

Appendix C.3. Study of different vascularizations with tumor and DCE in-silico images

Section 3.1.2 studies vascularized tumor cases for various MVDs reflected in the parameter value of n defined in equation B.5) based on Fig.C.12.

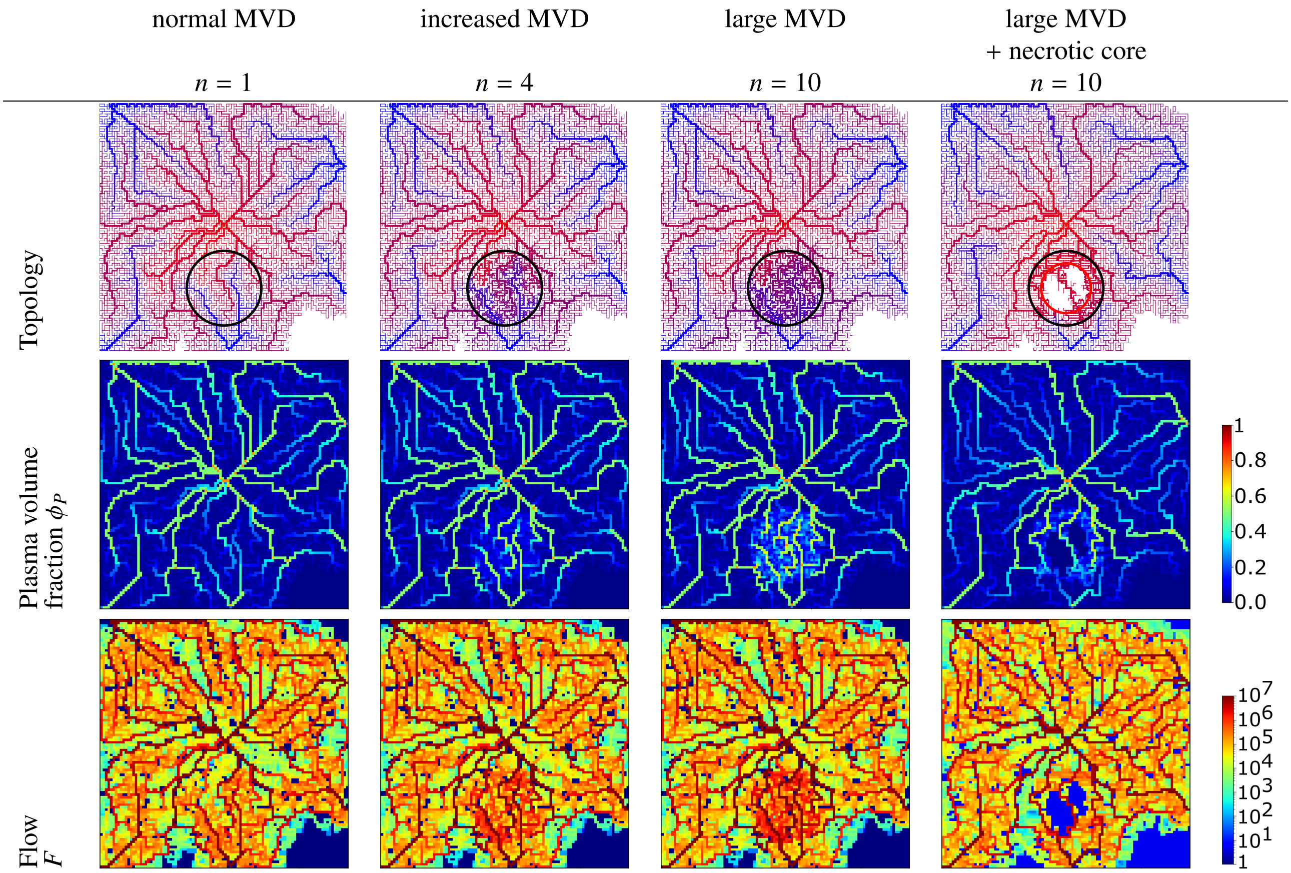

Figure C.12: **Different vascularizations**: healthy (left), increasing MVD (center columns) and with in addition a necrotic core (right). Top: connected arterial (root node at the center) and venous trees, colored by pressure (from higher pressure in red, to low pressure in blue). Black circles represent the tumor zone, and the red circle the necrotic region. Middle: plasma volume fraction, in each tissue voxel. Bottom: flow in $\mu m^3/s$. Domain: $6mmx6mmx60\mu m$.

Different tumor features translate into changes of in-silico perfusion images, see **Supplementary movies** with the following names:

- Variation of MVD (4, 20, 30) with no permeability: Permeability0MVD4.mpeg, Permeability0MVD20.mpeg, Permeability0MVD30.mpeg.
- Increase of MVD vs permeability in the tumor zone compared to the reference case (movies corresponding to Fig.4): Permeability0.1MVD30.mp4, Permeability0.1Baseline.mp4, Permeability0.1Tumor100.mp4.
- Variation of vascular rim at fixed tumor size (and thus necrotic core size): NONecroticCore.mpeg, SMALL-NecroticCore.mpeg, LARGENecroticCore.mpeg.

Appendix C.4. Inference: interpretation of recovered parameters - the one dimensional case

As the coarseness of the image resolution data and the lack of spatial consideration during the inverse procedure might lead to wrong conclusions, the aim of this section is to study the outcome of such an inverse procedure in a simple example, where the analytical solution of the concentration over time is known. As already introduced in section 3.2.1, consider a one-dimensional, straight piece of tissue of section $L*L$=60μm*60μm with a straight capillary inside of constant radius $r = 4\mu m$ (thus $\phi_P \approx 0.014$) of length 6mm (see Fig.C.13), containing an intravascular agent that is entering at the inlet (at $x = 0$) with the known concentration $C_A(t)$ taken here equal to the AIF. In that case, the plasma concentration is analytically given by equation (15).

(a) Considering this tissue as a given region of interest (ROI) of length l and volume $V = L^2 * l$, the average concentration in that volume is $\bar{c}(t) = \frac{1}{V}\int_\Omega c(x,y,z,t)dV = \frac{r^2\pi}{L^2}\frac{1}{l}\int_0^l c_p(x,t)dx = \frac{1}{l}\int_0^l c(x,t)dx$ (whereby $c(x,t) = c_p(x,t)\Theta(r^2 - y^2 - z^2)$, y, z the coordinate directions perpendicular to the orientation of the pipe, $\Theta(.)$ is the Heaviside function, and $\phi_P = \frac{r^2\pi}{L^2}$). Conservation of mass gives that it varies according to the flux of compound in and out of that volume:

$$\frac{d\bar{c}}{dt} = \frac{d(\phi_P \bar{c}_P)}{dt} = \frac{F}{V}(C_A(t) - c_P(x = l,t)) \tag{C.1}$$

The last equation is precisely the integrated version of equation (1) along the tissue length. Let us now take several ROI sizes and study how the different parameters are recovered in different settings (Fig.C.13a). First we will consider measurements which consist in both the ROI concentration $\bar{c}$ and the outlet measurement $c_P(x = l,t)$ (or equivalently $c(x = l,t)$) at the end of the ROI. Then taking equation C.1 as the model to estimate the two unknown parameters ϕ_P and F, those parameters are exactly recovered (Fig.C.13a). This is because the model to estimate the parameters is exactly equation C.1, the integrated form of equation 1, and corresponds to the conservation of mass in that ROI.

(b) In practice, one has access only to the average tissue measurement within an ROI. In that case a measurement of the ROI plasma concentration is often approximated by $\bar{c}_P(t) \approx (c_P(x = 0,t) + c_P(x = l,t))/2 = (C_A(t) + c_P(x = l,t))/2$, i.e. by the mid-point rule which then is considered a good approximation of the space-integral. Now taking Brix II model (equation 9) (assuming the exchange-rate is 0) to estimate the parameters, the recovered flow is 2 times higher than it should (Fig.C.13b). The derivation is performed in the paragraph below. This factor 2 is actually the limit case of the tissue-specific factor between the output vein concentration and the average capillary network concentration as found in Appendix of Brix et al. (2009).

The measurement taken to estimate the outlet concentration corresponds to the measurement of the contrast agent in the middle of the tissue sample, meaning as if the input contrast agent instead of having already travelled through the entire volume, had by definition of the midpoint only passed through half of the volume, or equivalently since the ROI volume is fixed, the flow is treated as if it was going two times faster. This corresponds to l/v (or $\phi_P V/F$) ≈time width of CA peak.

Factor of two. To explain the counter-intuitive result that the recovered flow is twice the original value when a measurement of the ROI plasma concentration $\bar{c}_P$ and flows for which $\bar{c}_P(t) \approx (C_A(t) + c_P(x = l,t))/2$ (i.e. the mid-point rule is a good approximation of the space-integral) is considered, we here derive the underlying plasma flow equation.
Starting from conservation of mass (equation C.1):

$$\frac{d(\phi_P \bar{c}_P)}{dt} = \frac{F}{V}(C_A(t) - c_P(x = l,t))$$

and replacing $c_P(x = l, t)$ from $\bar{c}_P(t) \approx (C_A(t) + c_P(x = l, t))/2$ by $2\bar{c}_P(t) - C_A(t)$, one gets:

$$\frac{d(\phi_P \bar{c}_P)}{dt} = \frac{2F}{V}(C_A(t) - \bar{c}_P(t))$$

This is Brix II model (equation 9) (assuming the exchange-rate is 0), up to a factor 2 for the flow. Hence, using this pharmacokinetic model to estimate the flow F, the recovered flow is twice the true value.

(c, d) Now the ROI does not necessarily contain the point of feeding of the contrast-agent, which has already been considered in sect. 3.2.1. Here, multiplying the recovered flow F_{recov} with $N_i = x/l_i$, leads to the correct flow $F = F_{recov} \times N_i$ (Figs. 5a1, C.13c, d).

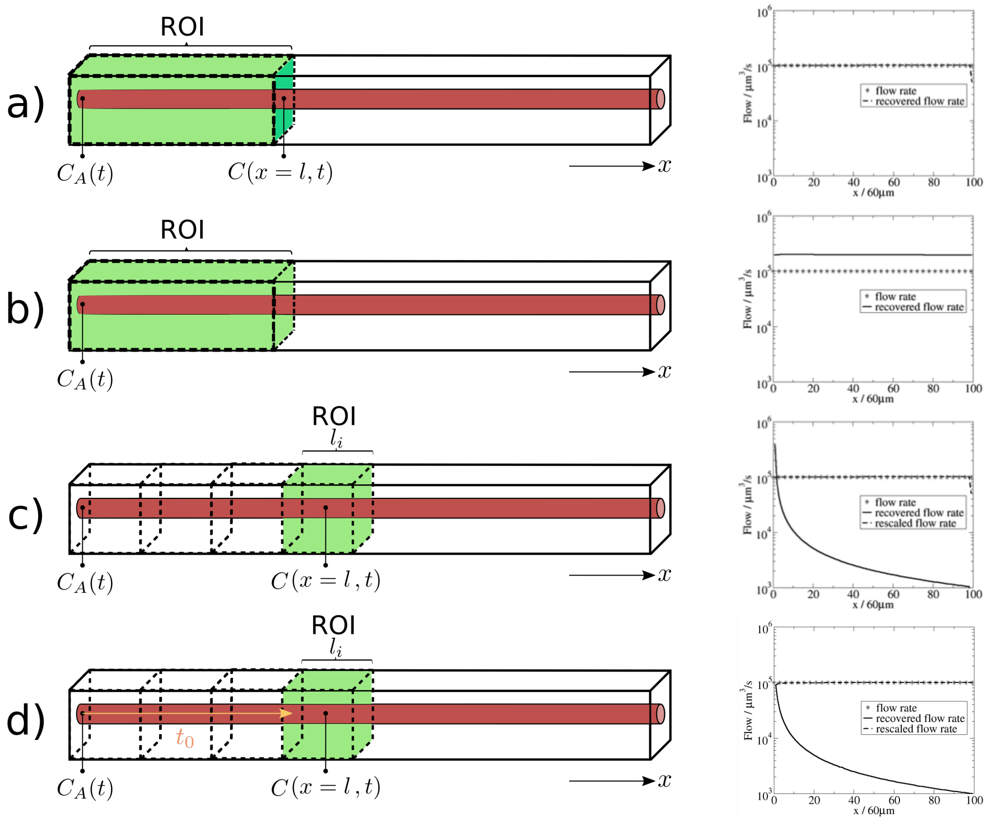

Figure C.13: **Meaning of flow in inverse models.** Legend: 'flow rate' is the ground truth, 'recovered flow rate' is the estimated flow by the inverse procedure, 'rescaled flow' is the estimated flow multiplied by $N_i = x/l_i$. Domain: 100 voxels of size each $(60\mu m)^3$. a) ROI includes inlet point and ROI-average and outlet concentrations are assumed to be known; Using Brix II model: b) ROI includes inlet point but outlet concentration is not known, c) ROI does not include inlet point. Using DE-Tofts: d) ROI does not include inlet point.

Appendix C.4.1. Small ROI

Conservation of mass in an ROI of length l that includes the feeding point reads as the above equation Appendix C.4:

$$\frac{d(\phi_P \bar{c}_P|_{0-l})}{dt} = \frac{F}{V_{0-l}}(C_A(t) - c_P(x = l, t))$$

Let us define $C_P(t)$ as the average concentration of $c(x, t)$ over a small ROI which ends at $x = l$ and of size $V_i = L^2 * l_i$. Since the ROI is small in the sense described before of $F/(\phi_P V) > 1$/time-width of CA peak, $c_P(x = l, t) \approx C_P(t)$ and

$V_{0-l} \approx N_i V_i$. Thus conservation of mass in the large ROI becomes:

$$\frac{d(\phi_P \bar{c}_P|_{0-l})}{dt} \approx \frac{F}{N_i V_i}(C_A(t) - C_P(t)).$$

If the model of Brix II,

$$\frac{d(\phi_P C_P)}{dt} = \frac{F_{recov}}{V_i}(C_A(t) - C_P(t)),$$

is used for the inference parameter of the small ROI given its average concentration $C_P(t)$, one recovers $F_{recov} \approx F/N_i$. Hence the need to rescale the recovered flow rate as explained in the results section.

Appendix C.4.2. Sensitivity to noise

To test the effect of noise in the measurements, a Gaussian white noise of standard deviation 5% and 20% of the time-averaged value for zero noise, $\frac{1}{T}\int_0^T c(t; \sigma = 0)dt$, is added to the tissue marker concentration in the first 1d test case above, which defines the zero noise ($\sigma = 0$) case (Fig.C.14 right). The error in the tissue measurement (top) affects much more how flow is estimated (middle) compared to the plasma volume fraction (bottom), which is always well estimated: for 5% noise, the flow is estimated up to a factor of 2, but for 20%, this is still true only when sufficiently far from the inlet.

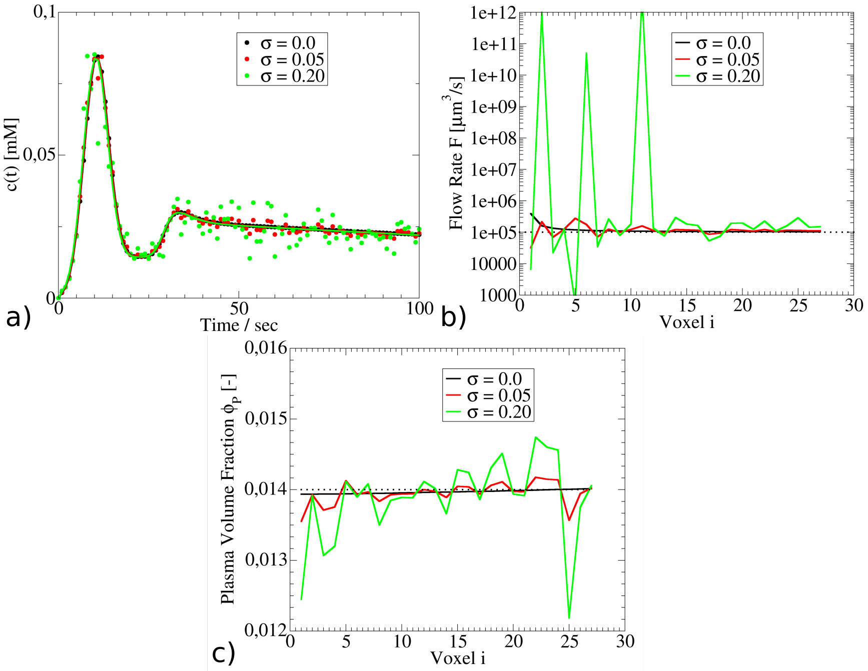

Figure C.14: **Sensitivity of the inverse procedure to noise.** The AIF was feeding a 1d tissue slab that contains a vessel like in (Fig.C.13c). The legend indicates the level of added noise, from 5% to 20% of time-averaged noise-free concentration. a) Resulting tissue concentration over time for different parameters (ROI average), b) Rescaled flow rate, c) Plasma volume fraction ϕ_P. For the flow (b) and ϕ_P (c), the dotted lines correspond to the input parameters, while the original plasma volume fraction is 0.014 in all cases.

Appendix C.5. Intra-vascular agent in a multidimensional case

The case of intravascular agent propagation through a 2d microvasculature that contains a vascularised tumor has been discussed for the Brix II model in sect. 3.2.1. The direct comparison with the Brix II inference results shows that the DE-Tofts model behaves very similar as the Brix II model (Fig.C.15). The only difference is the estimated exchange rate in regions where the plasma volume fraction $\phi_p = 0$, which however is irrelevant as no vessels can be situated in those zones in case of zero vessel permeability (this difference is found for all studied permeabilities, compare Figs. 6, 7).

Appendix C.6. Inferred tissue parameter maps for zero permeability in 2d

Fig. (C.15) shows all the recovered parameters for Brix II and DE-Tofts when $P = 0\mu m/s$ corresponding to the summary Fig.5 of the main text.

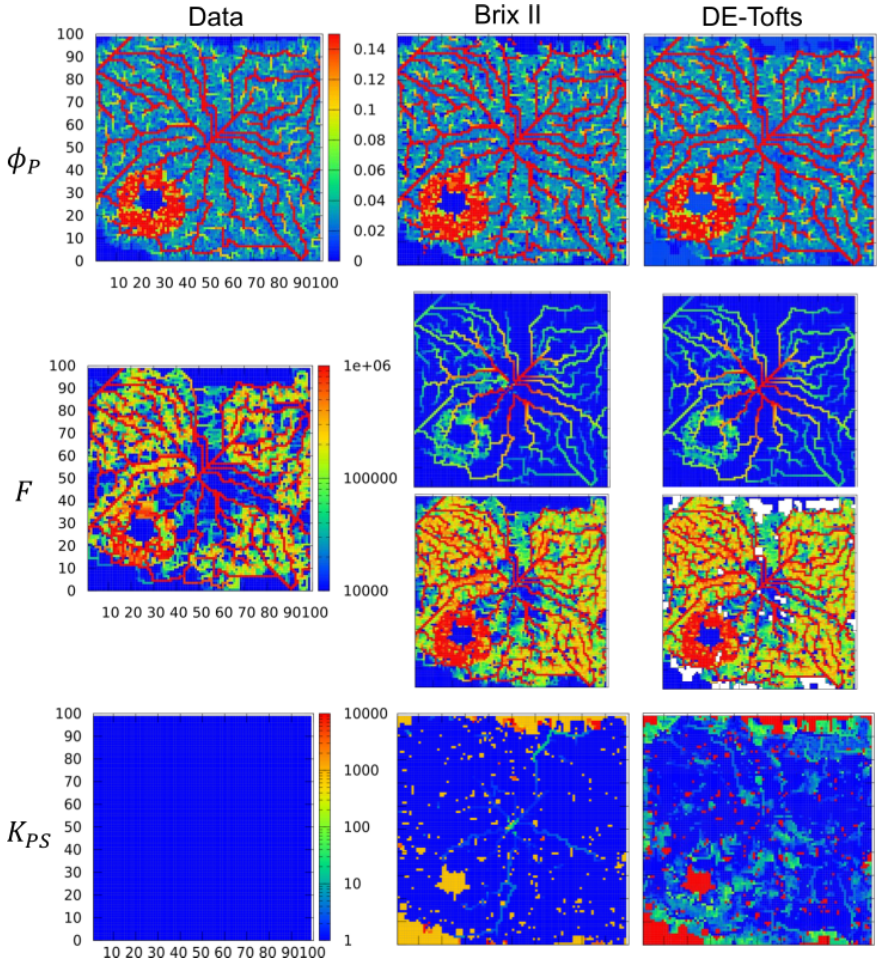

Figure C.15: **Inferred TK model parameters from contrast-agent time course in 2d.** Columns: left=data, center=Brix II, right=DE-Tofts, vessels are non-permeable: $P = 0$. First row: vascular density ϕ_P, second row: flow, third row: rescaled flow, fourth row: exchange rate K_{PS}. Voxel size: $(60\mu m)^3$, domain: 100x100x1 voxels. F and K_{PS} in $\mu m^3/s$. Same scale bar for all ϕ_P,F,K_{PS}.

Appendix C.7. Large ROI and 3d parameters map

Fig. (C.16) shows comparison for ϕ_P and F for Brix II and DE-Tofts when $P = 0\mu m/s$ corresponding to the summary Fig.5 of the main text.

Appendix C.8. Extra-vascular agents - the one dimensional case

The recovery of parameters when the contrast-agent can extravasate is studied in section 3.2.2, showing in 1d results of the concentration curve fit in Fig.C.17 and the parameter recovery in Fig.C.18.

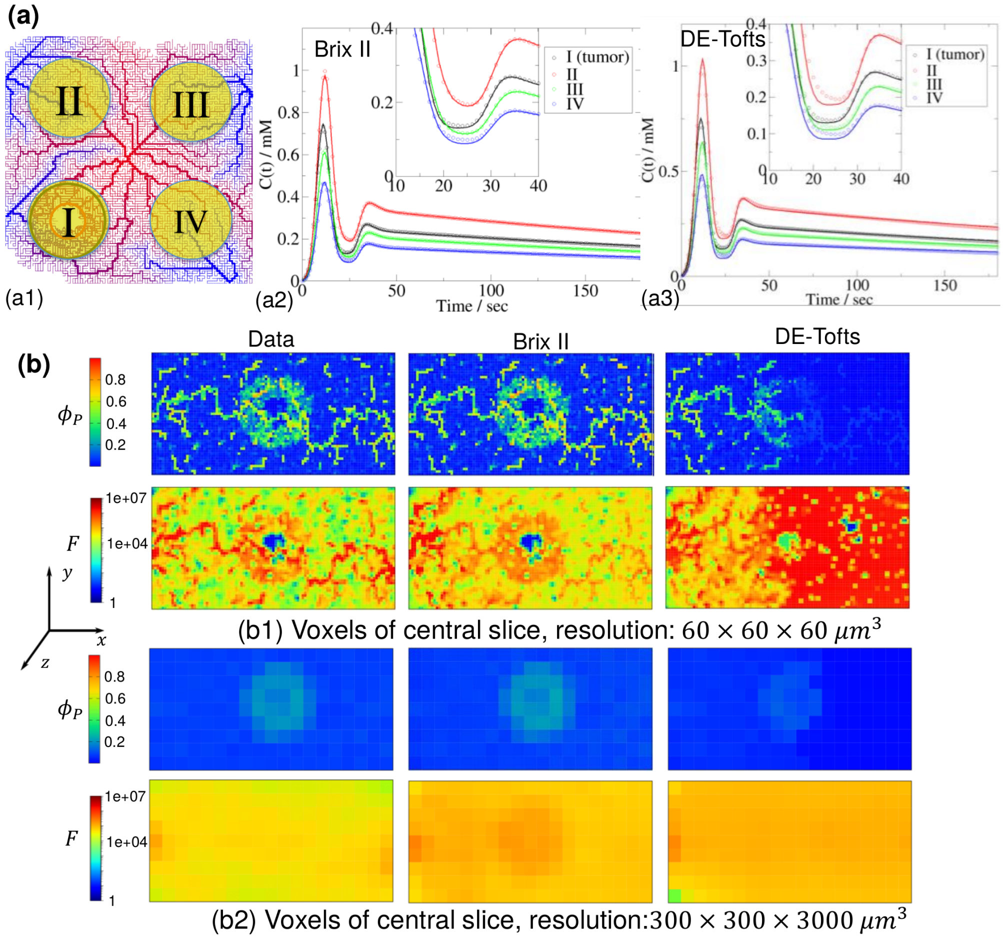

Figure C.16: **Large ROI (a) and 3d case (b) parameter inference: Brix II vs DE-Tofts comparison** (a1): vascularization topology (from high (red) to low (blue) pressure) and 4 different regions of interest (ROI size=709 voxels, ≈ 2mm diameter). I contains the tumor necrotic zone (red circle) inside the tumor rim (green circle). Feeding artery in the center of the image. (a2, a3): the fit (continuous curves) of the measured contrast-agent concentrations over time (circles) for the four zones by the Brix II and DE-Tofts models. (b) Parameter inference from 3d data ($6mm \times 3mm \times 3mm$). The feeding artery is on the left of the images (central slices in z-direction). (b1) ϕ_p (1rst row) and flow (2nd row) for a $(60\mu m)^3$ resolution; (b2) ϕ_p (1rst row) and flow (2nd row) for a $300 \times 300 \times 3000 \mu m^3$ resolution. Columns: data (left), Brix II. (center), DE-Tofts (right). F and K_{PS} in $\mu m^3/s$.

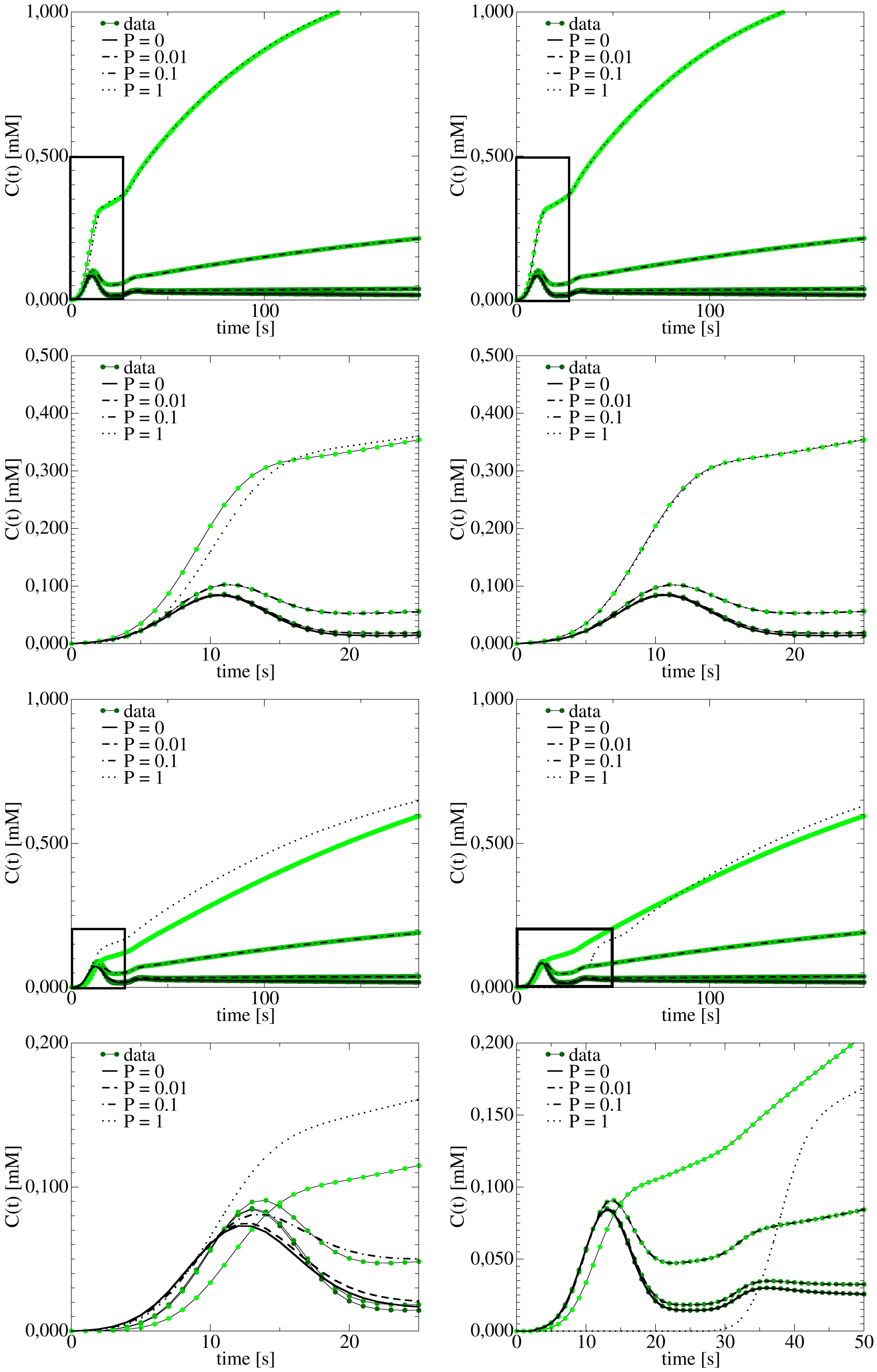

Figure C.17: **Fit of TK models to contrast-agent time course in 1d.** Left=Brix II, right=DE-Tofts. Plasma concentration at voxel 10 (first row), zooms for early times in squared region (second row), at voxel 90 (third row) and corresponding zooms in early times (fourth row). P $(\mu m/s)$ is the permeability coefficient. Green curves denote the original data.

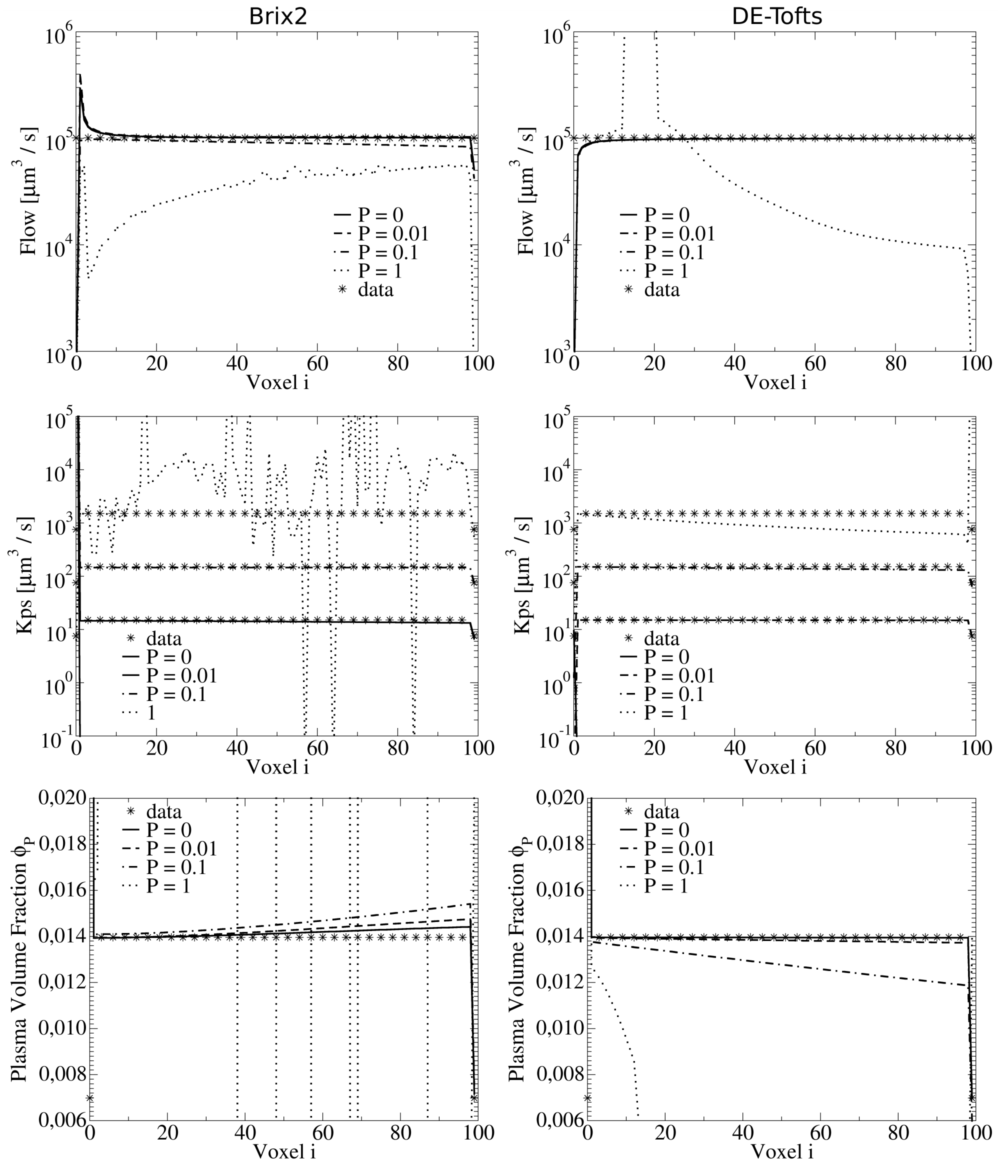

Figure C.18: **Inferred TK model parameters from contrast-agent time course in 1d.** Left=Brix II, right=DE-Tofts. First row = rescaled flow, second row = exchange rate Kps, third row = vascular density ϕ_P. P is in $\mu m/s$.Voxel size: $(60\mu m)^3$, domain:100 voxels.
